# Ecological and evolutionary processes involved in shaping microbial habitat generalists and specialists in urban park ecosystems

**DOI:** 10.1128/msystems.00469-24

**Published:** 2024-05-20

**Authors:** Shuzhen Li, Xue Yan, Mamun Abdullah Al, Kexin Ren, Christopher Rensing, Anyi Hu, Andrey N. Tsyganov, Yuri Mazei, Alexey Smirnov, Natalia Mazei, Jun Yang

**Affiliations:** 1 Aquatic EcoHealth Group, Fujian Key Laboratory of Watershed Ecology, Key Laboratory of Urban Environment and Health, Institute of Urban Environment, Chinese Academy of Sciences, Xiamen, China; 2 University of Chinese Academy of Sciences, Beijing, China; 3 Institute of Environmental Microbiology, College of Resources and the Environment, Fujian Agriculture & Forestry University, Fuzhou, China; 4 CAS Key Laboratory of Urban Pollutant Conversion, Institute of Urban Environment, Chinese Academy of Sciences, Xiamen, China; 5 Lomonosov Moscow State University, Moscow, Russia; 6 Faculty of Biology, Shenzhen MSU-BIT University, Shenzhen, China; 7 A.N. Severtsov Institute of Ecology and Evolution, Russian Academy of Sciences, Moscow, Russia; 8 Department of Invertebrate Zoology, Faculty of Biolog, St. Petersburg University, St Petersburg, Russia; Third Institute of Oceanography Ministry of Natural Resources, Xiamen, China

**Keywords:** microbial communities, urban ecosystems, prokaryote, microeukaryote, microbial interactions, microbial diversity

## Abstract

Microbiomes are integral to ecological health and human well-being; however, their ecological and evolutionary drivers have not been systematically investigated, especially in urban park ecosystems. As microbes have different levels of tolerance to environmental changes and habitat preferences, they can be categorized into habitat generalists and specialists. Here, we explored the ecological and evolutionary characteristics of both prokaryotic and microeukaryotic habitat generalists and specialists from six urban parks across five habitat types, including moss, soil, tree hole, water, and sediment. Our results revealed that different ecological and evolutionary processes maintained and regulated microbial diversity in urban park ecosystems. Under ecological perspective, community assembly of microbial communities was mainly driven by stochastic processes; however, deterministic processes were higher for habitat specialists than generalists. Microbial interactions were highly dynamic among habitats, and habitat specialists played key roles as module hubs in intradomain networks. In aquatic interdomain networks, microeukaryotic habitat specialists and prokaryotic habitat specialists played crucial roles as module hubs and connectors, respectively. Furthermore, analyzing evolutionary characteristics, our results revealed that habitat specialists had a much higher diversification potential than generalists, while generalists showed shorter phylogenetic branch lengths as well as larger genomes than specialists. This study broadens our understanding of the ecological and evolutionary features of microbial habitat generalists and specialists in urban park ecosystems across multi-habitat.

**IMPORTANCE:**

Urban parks, as an important urban greenspace, play essential roles in ecosystem services and are important hotspots for microbes. Microbial diversity is driven by different ecological and evolutionary processes, while little is currently known about the distinct roles of ecological and evolutionary features in shaping microbial diversity in urban park ecosystems. We explored the ecological and evolutionary characteristics of prokaryotic and microeukaryotic habitat generalists and specialists in urban park ecosystems based on a representative set of different habitats. We found that different ecological and evolutionary drivers jointly maintained and regulated microbial diversity in urban park microbiomes through analyzing the community assembly process, ecological roles in hierarchical interaction, and species diversification potential. These findings significantly advance our understanding regarding the mechanisms governing microbial diversity in urban park ecosystems.

## INTRODUCTION

Urban parks, as urban green spaces that have increased significantly in recent decades, provide many essential services including water and air purification, wind and noise reduction, microclimate regulation, and social and psychological well-being by supporting ecosystem functioning in cities (1–3). Urban parks provide essential habitats for a wide range of species, promoting urban biodiversity and supporting ecosystem resilience. Prokaryotes and microeukaryotes are the key components of urban ecosystems that perform many important ecosystem functions, including nutrient cycling, plant growth regulation, and maintenance of ecosystem stability (4). Prokaryotes and microeukaryotes are highly connected through microbial food webs (5) and have ecological interactions with human health. Although microbial communities have vital roles in the urban environment, their diversity profiles in urban parks have rarely been explored (6, 7). A previous study has shown that ecological and evolutionary processes simultaneously regulate microbial diversity, but the mechanisms were shown to work in different ways (8). For example, ecological processes, including environmental filtering and species interactions, greatly affect diversity, while diversity originating from evolutionary processes is constrained by speciation and extinction rates (8). To date, evolutionary processes of microbial communities have largely been unexplored compared with ecological processes (9). The accelerating urbanization can lead to rapid changes in urban environments, breaking down a stable ecosystem state and creating new ecological niches, and may result in different evolutionary and ecological characteristics compared with natural ecosystems. Limited attention and focuses on a single habitat in the past studies, primarily in soil, have resulted in fragmented views of urban microbiomes and their diversity maintenance (2, 6, 7). This hinders the preservation and sustainable management of urban parks, which are crucial for promoting urban sustainability, biodiversity conservation, and human well-being in an increasingly urbanized world. Therefore, urban park ecosystems that integrate diverse habitats (i.e., moss, sediment, soil, tree hole, and water), along with their microbiomes (i.e., prokaryotes and microeukaryotes) are valuable research objects to enhance our understanding of microbial diversity patterns, and underlying ecological and evolutionary features regulating them.

Microbes can be classified as habitat generalists and specialists depending on their niche breadth and distribution (5). Habitat generalists are remarkably ubiquitous with wide habitat preferences, whereas specialists are specific to certain habitats displaying narrow environmental tolerances (10). Studies have revealed that the diversity of microbial habitat generalists and specialists is driven by different ecological and evolutionary processes. For example, community assembly processes (deterministic and stochastic processes) are important drivers of microbial diversity and composition (5, 11). Deterministic factors govern community structure through species traits, interspecies interactions, and local environmental conditions, while stochastic factors regulate community through speciation, extinction, colonization, birth, and death (12). Studies have shown that deterministic processes played more important roles in shaping the microeukaryotic generalists and specialists than those of prokaryotes in urban and riverine ecosystems (5, 13). Moreover, stochastic and deterministic processes significantly dominated bacterial generalist and specialist assemblages, respectively (11, 14, 15). These inconsistent observations indicate the necessity of comprehensive examinations of several microbial components regarding various habitat types. Furthermore, molecular ecological networks can represent various biological interactions, including mutualism, competition, and predation (16). The complex microbial interdomain (e.g., associations between prokaryotes and microeukaryotes) and intradomain (e.g., associations within prokaryotes or microeukaryotes) interactions are essential for maintaining community diversity and ecosystem functions (17). Recent studies have found that bacterial and microeukaryotic specialists play a significant role in maintaining network structure by acting as keystone species while comparatively a few habitat generalists could contribute for that (5, 14). Under an evolutionary perspective, speciation and extinction rates greatly shape microbial diversity (10). Moreover, studies of prokaryotic generalists and specialists on large spatial scales suggest that different evolutionary characteristics in multiple survival strategies might be responsible for maintaining microbial diversity, and inconsistent evolutionary directions between generalists and specialists have been observed (10, 15, 18). The urban park ecosystem offers a unique setting that may affect the evolutionary trajectories of species in ways that differ from other environments. Revealing these patterns can provide insights into the complex interactions between microbes and their urban environments and potentially enhance conservation and management strategies. However, little is known on how ecological and evolutionary features maintain microbial diversity and community assembly of habitat generalists and specialists in urban parks.

To address the knowledge gaps, we employed high-throughput sequencing approaches for the 16S and 18S rRNA genes to investigate prokaryotes and microeukaryotes from five types of habitats (moss, sediment, soil, tree hole, and water) in urban park ecosystems, respectively. This study aimed to (i) disentangle the diversity patterns of habitat generalists and specialists in urban park ecosystems and (ii) explore the ecological and evolutionary characteristics of habitat generalists and specialists and their distinct roles in maintaining microbial diversity patterns and community assembly. We hypothesized that (i) there are distinct community compositions of habitat generalists and specialists among habitats, with specialists exhibiting higher richness and relative abundances compared with generalists. (ii) Both ecological and evolutionary processes are crucial for generating and maintaining biodiversity. Deterministic processes hold greater significance for habitat specialists due to their relatively weaker adaptability to the environment compared with generalists. The significance of interspecific interactions and species diversification of specialists surpasses that of generalists, resulting in specialists dominating the majority of biodiversity.

## RESULTS

### Diversity pattern of habitat generalists and specialists

**Fig 1:**
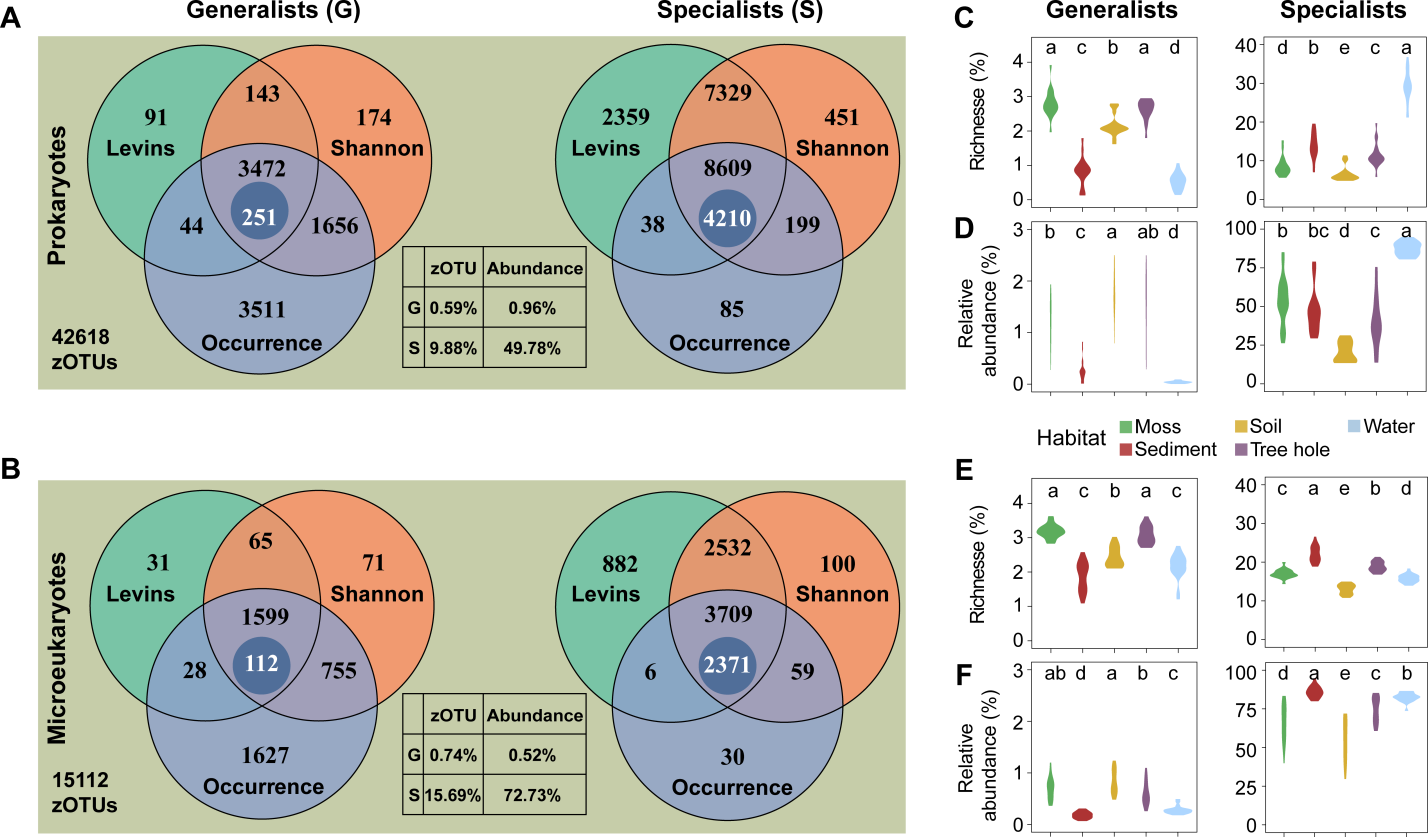
Habitat generalists and specialists of (**A**) prokaryotes and (**B**) microeukaryotes, respectively. Numbers in the Venn diagram represent the generalists and specialists identified by three methods, namely, Levins’ niche breadth, Shannon diversity, and occurrence frequency. Only the zOTUs shared by three methods are considered as habitat generalists or specialists. Generalists from the upper 10th percentile of all three-index values are considered as strict generalists, and specialists with over 50 reads are defined as strict habitat specialists in this study. Only these strict generalists and specialists are retained for subsequent analysis. The deep blue circle with a number inside defines strict habitat generalists and specialists. Values in the table are percentages of zOTUs and relative abundances of strict habitat generalists and specialists, respectively. Richness and relative abundance of (**C, D**) prokaryotic and (**E, F**) microeukaryotic generalists and specialists in five urban park habitats. Different letters above the violin plot indicate significant differences between habitats at *P* < 0.05.

A total of 90 samples from moss, sediment, soil, tree hole, and water habitats were collected in six urban parks to explore the ecological and evolutionary properties of microbial habitat generalists and specialists (Fig. S1; Tables S1 and S2). For prokaryotes and microeukaryotes, the rarefaction curves of most samples almost flattened (Fig. S2), and all samples were found to have a high Good’s coverage (>0.9) (Table S3). These results indicated that the sequencing depth was sufficient to capture most of the microbial diversity. Overall, there were 42,618 and 15,112 prokaryotic and microeukaryotic zero-radius operational taxonomic units (zOTUs), respectively (Fig. 1). Among them, 251 (0.59%) strict habitat generalist zOTUs and 4210 (9.88%) strict habitat specialist zOTUs were identified, and they represented 0.96% and 49.78% of the total sequences for prokaryotes, respectively (Fig. 1A). Similarly, 112 (0.74%) strict habitat generalist zOTUs and 2371 (15.69%) strict habitat specialist zOTUs were identified for microeukaryotes with relative abundances of 0.52% and 72.73%, respectively (Fig. 1B). The richness and relative abundance of habitat generalists and specialists showed significant differences across the five habitats, with a significantly higher number of both prokaryotic and microeukaryotic habitat generalists found in moss followed by tree hole (Fig. 1C and E), and a higher relative abundance found in soil (Fig. 1D and F). In contrast, a higher number and relative abundance of prokaryotic specialists were found in water (Fig. 1C and D), whereas sediment exhibited a higher number and relative abundance of microeukaryotic specialists (Fig. 1E and F).

Most of the prokaryotic phyla consisted of specialists and opportunists; notably, habitat specialists accounted for more than 70% of the relative abundance for microeukaryotic phyla (Fig. S3). Different dominant taxa showed distinct preferences for generalists or specialists across five habitats (Fig. S4). For instance, the prokaryotic taxa Proteobacteria and Actinobacteriota were more dominant for generalists, while Cyanobacteria and Bacteroidetes were more dominant for specialists (Fig. S4A). As for microeukaryotes, Cercozoa, Chlorophyta, Ciliophora, Fungi, and Metazoa were dominant for both generalists and specialists (Fig. S4B). Additionally, rare taxa, mainly Ochrophyta, were only observed as generalists, whereas Conosa, Dinoflagellata, and Streptophyta tended to be specialists (Fig. S4B).

The hierarchical cluster analysis and permutational multivariate analysis of variance (PERMANOVA) tests demonstrated that samples were more clustered by habitat type rather than park, indicating a significant effect of habitat type on urban microbiomes (Fig. S5; Table S4). Results from NMDS and PCoA analyses further indicated that habitat specialists greatly varied among the five habitats (Fig. S6 and S7). This variation was much higher for habitat specialists (*R* = 0.764 and 0.829 for prokaryotes and microeukaryotes, respectively) than generalists (*R* = 0.382 and 0.420 for prokaryotes and microeukaryotes, respectively) based on Bray-Curtis dissimilarity (Fig. S6). Meanwhile, habitat generalists were much more clustered in terrestrial habitats (i.e., soil, moss, and tree hole) than in aquatic ones (i.e., water and sediment) (Fig. S6 and S7). Community dissimilarity significantly increased with geographical distance both for taxonomic and phylogenetic resolutions (Fig. S8 and S9), and the turnover rate was consistently lower for generalists compared with specialists, suggesting generalists might easily adapt and survive across various habitats or parks.

### Community assembly processes of habitat generalist and specialist

**Fig 2:**
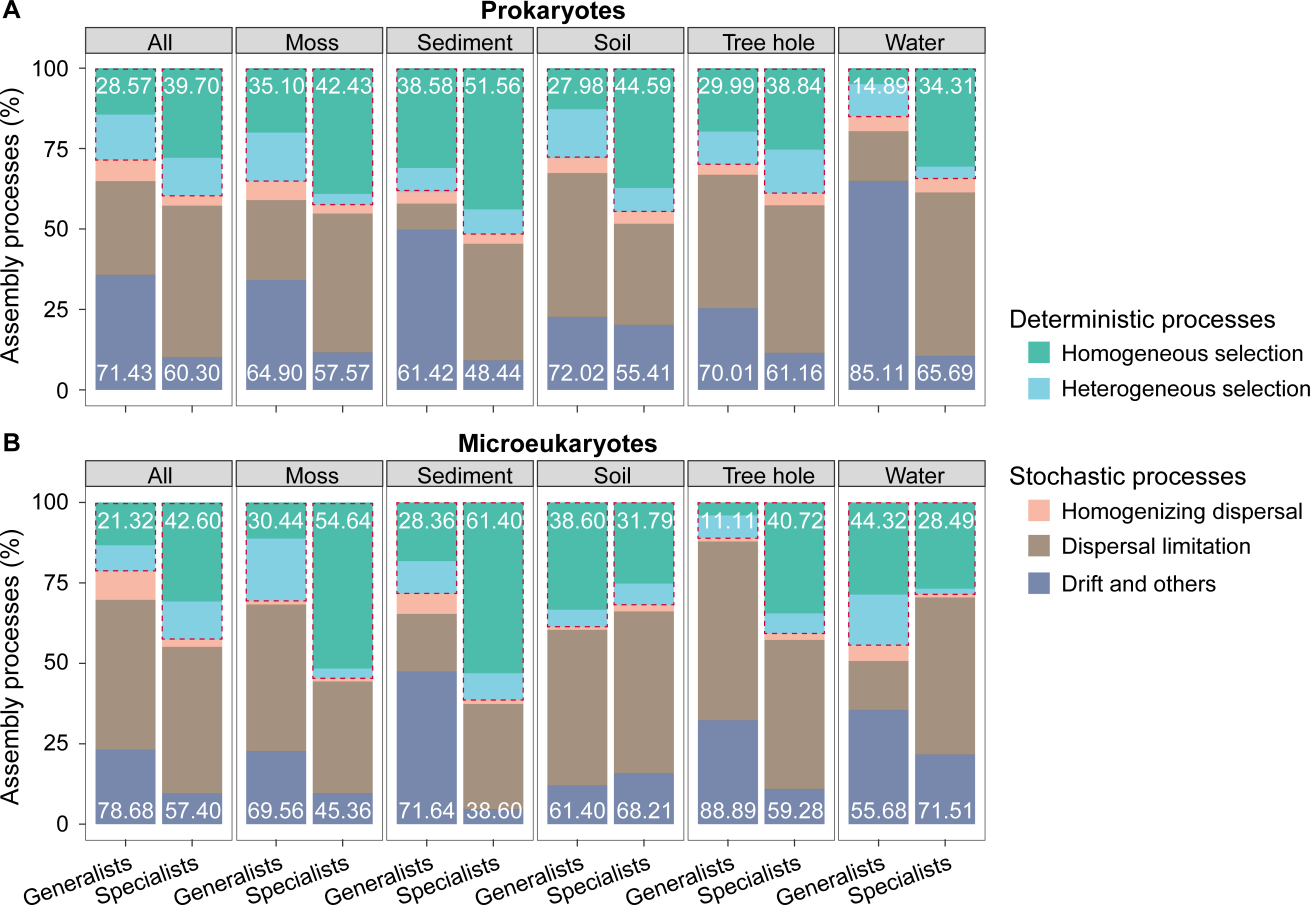
Community assembly processes of (**A**) prokaryotic and (**B**) microeukaryotic generalists and specialists, respectively. The deterministic processes include both homogeneous selection and heterogeneous selection, and the stochastic processes include homogenizing dispersal, dispersal limitation, and drift and others. The part enclosed by the dashed line represents the deterministic process. The numbers at the upper and lower ends of the bar chart represent the total contribution of deterministic and stochastic processes to the community assembly, respectively. “All” represents the result from all five habitats.

Five processes were assessed in governing community assembly through a null model (Fig. 2). Stochastic processes contributed approximately 60.30%–71.43% for prokaryotic communities and around 57.40%–78.68% for microeukaryotic communities, indicating that stochastic processes dominated community assembly in urban park ecosystems. Overall, dispersal limitation showed significant contributions for generalists and specialists, revealing restricted microbial movement within habitats. Drift played a more significant important role in generalists compared with specialists (except for microeukaryotes in soil), and specialists were more influenced by dispersal limitation, particularly in aquatic environments (i.e., water and sediment). Additionally, the relative importance of deterministic processes, especially homogeneous selection, was stronger for habitat specialists, except for microeukaryotes dwelling in soil and water, indicating community compositions of specialists were more similar under homogeneous environmental conditions.

### Intradomain and interdomain ecological networks

**Fig 3:**
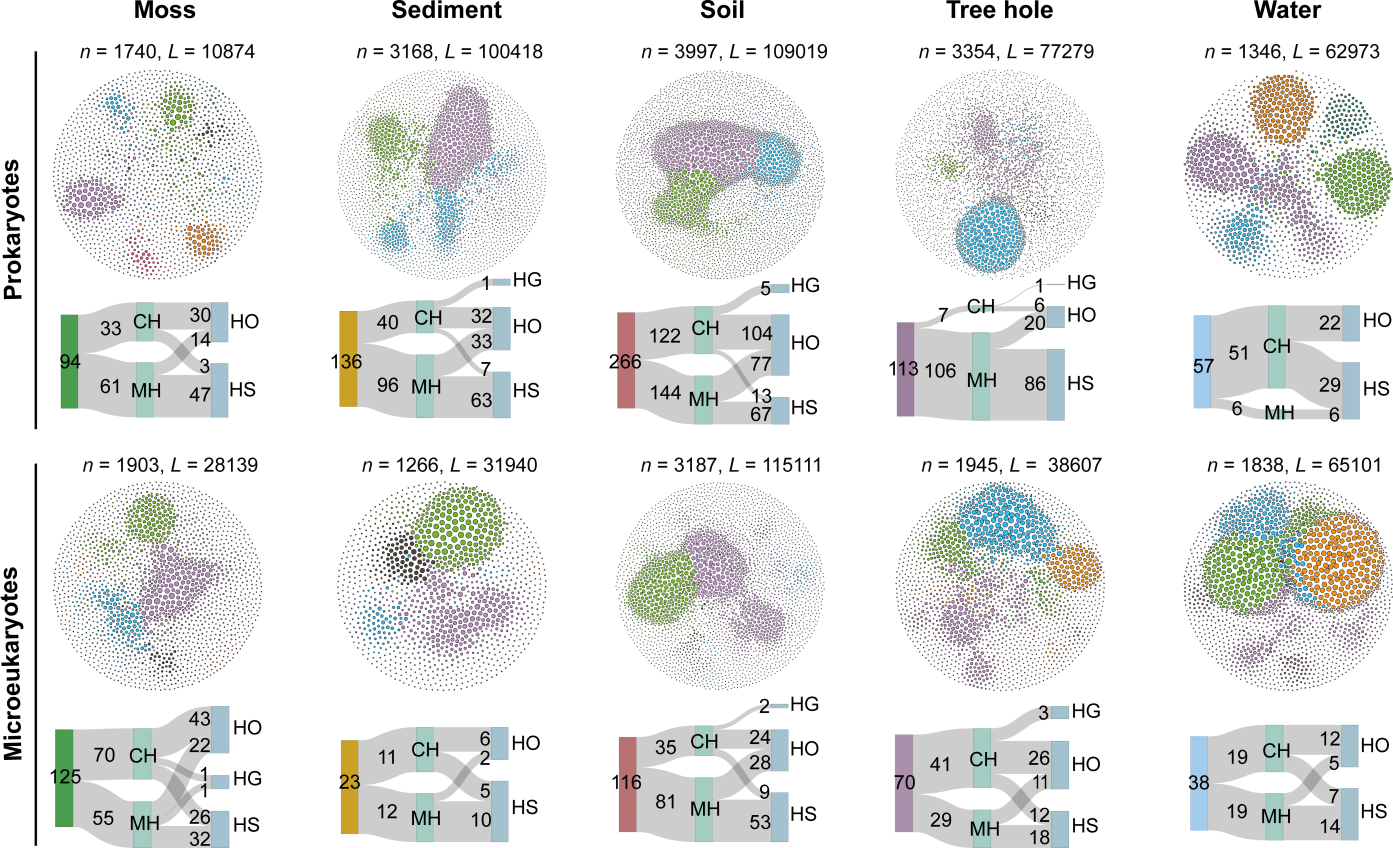
Molecular ecological networks of prokaryotes and microeukaryotes in five urban park habitats, showing the number of keystone taxa of habitat generalists and specialists. Nodes are colored based on different modules. Node size is proportional to the degree, with larger node indicating higher degrees. *n* and *L* represent the node number and link number, respectively. Keystone taxa are identified based on within-module connectivity and among-module connectivity. The connector hub (CH) is highly linked to other modules with high among-module connectivity, and the module hub (MH) is highly linked to numerous nodes within its own module with high within-module connectivity. The Sankey plots show the number of identified keystone species, including CH and MH, for habitat generalists (HG), habitat specialists (HS), and other species (i.e., habitat opportunists [HO]).

To explore potential ecological interactions between species, we constructed molecular ecological networks within each local habitat. These constructed networks exhibited a good fit with the power-law model (*R*^2^ > 0.9 except for the prokaryotic network in water), indicating a scale-free characteristic where most nodes have few neighbors while few nodes have a large number of neighbors (Table S5). Network topological properties from the empirical network (i.e., average clustering coefficient and modularity) were significantly higher than those of the randomized networks for both prokaryotes and microeukaryotes, implying a modular structure in the networks with some nodes highly connected to their neighbors. Microbial interactions were highly dynamic as the network size displayed great variations among habitats, ranging from 1,346 to 3,997 and 1,266 to 3,187 nodes for prokaryotes and microeukaryotes, respectively (Fig. 3; Table S5). We found much higher positive interactions than negative ones, and soil harbored the most nodes and links, but the lowest modularity. To facilitate the comparison of the roles of specialists and generalists and to eliminate the impact of varying species numbers, we specifically focused on comparing the proportions of specialists or generalists in the network to total specialists or generalists, respectively (Fig. S10). There were comparable proportions of specialists and generalists in terrestrial ecosystems (i.e., moss, soil, and tree hole; Fig. S10A), and much higher percentages of specialists than generalists were found in aquatic environments for both prokaryotes (37.16% vs. 3.72% in sediment and 44.28% *vs*. 0% in water; Fig. S10A) and microeukaryotes (44.68% vs. 11.32% in sediment and 56.73% *vs*. 22.94% in water; Fig. S10A), indicating specialists were more connected to microbial network interaction.

In addition to the network topological properties, network structure differed between habitats (Fig. 3). Two kinds of keystone species (connectors and module hubs) were identified in this study. Connectors are highly linked to other modules with high among-module connectivity, and module hubs are highly connected to many nodes within its own module with high within-module connectivity. For prokaryotes, soil (266) and water (57) harbored the highest and lowest numbers of keystone species, while for microeukaryotes, moss (125) and sediment (23) had the most and least keystone species. Habitat generalists rarely played a major role in biological interaction; in contrast, specialists were extremely important as module hubs in most habitats (Fig. 3). The percentages of identified keystone specialists to total specialists ranged from 1.80% to 3.17% for prokaryotes and from 1.16% to 3.68% for microeukaryotes, which were higher than those of generalists in most habitats (Fig. S10B).

The simplified networks which exclusively comprised prokaryotic and microeukaryotic habitat generalists and specialists demonstrated a significantly higher modularity compared with randomized networks, suggesting that the empirical interdomain networks were modular (Table S6). Prokaryotes and microeukaryotes displayed the highest robustness (the tolerance of the system to species extinctions) in water; moreover, sediment exhibited the highest functional complementarity (the total branch length of a functional dendrogram based on differences in visitor assemblages), implying a stable cross-domain species coexistence of microbes in aquatic environments. Notably, in moss and soil, microeukaryotes were more actively involved in interactions than prokaryotes, whereas prokaryotic nodes were higher in aquatic habitats compared with microeukaryotes. Overall, terrestrial habitats exhibited higher positive link proportions and module numbers than aquatic habitats. Soil had the most interaction links (33,123), while there was relatively less association for microbes in moss (1,243) and tree hole (1,916). A higher proportion of habitat specialists than generalists participated in interdomain interactions in four habitats other than soil (Fig. S10C).

**Fig 4:**
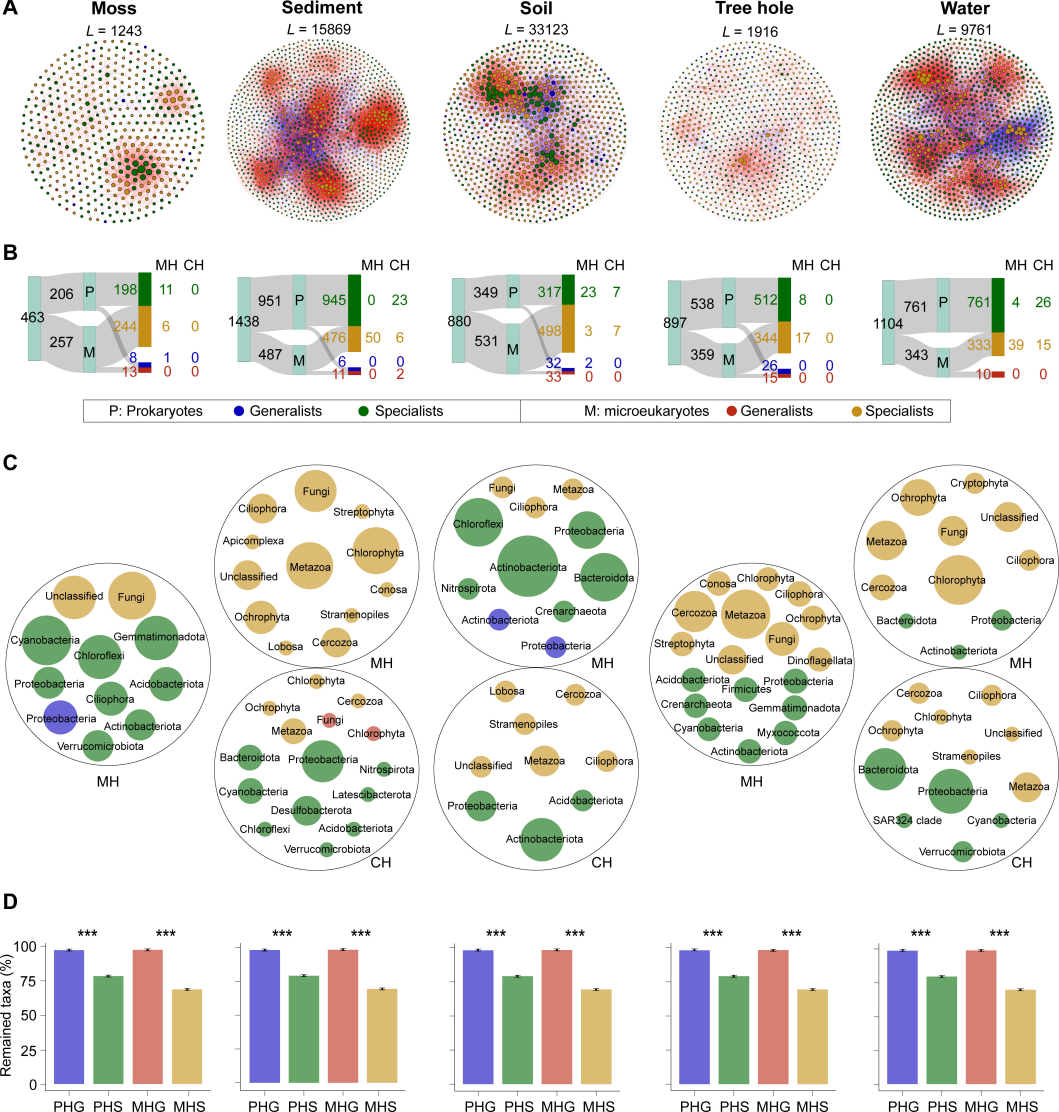
Interdomain networks from strict prokaryotic and microeukaryotic habitat generalists and specialists in five habitats in urban park ecosystems. (**A**) The modular association of the microbial communities. Nodes are colored based on prokaryotic and microeukaryotic habitat generalists and specialists. Node size is proportional to node degree, with larger node indicating higher degrees. *L* represents the link number. The red and blue edges represent positive and negative correlations, respectively. (**B**) The Sankey plots show the number of habitat generalists and specialists in the network, and corresponding MH and CH. Keystone taxa are identified based on within-module connectivity and among-module connectivity. CH is highly linked to other modules with high among-module connectivity, and MH is highly linked to numerous nodes within its own module with high within-module connectivity. P, prokaryotes; M, microeukaryotes. (**C**) The taxonomic annotation of identified keystone prokaryotes and microeukaryotes. Circle sizes are proportional to taxa number in each habitat. (**D**) The proportion of taxa remained with 50% of the habitat generalists or specialists randomly removed from each empirical network. The error bar represents the standard deviation of 100 repetitions at ***P < 0.001. PHG, prokaryotic habitat generalists; PHS, prokaryotic habitat specialists; MHG, microeukaryotic habitat generalists; MHS, microeukaryotic habitat specialists.

In terrestrial ecosystems, prokaryotes served as keystone in interdomain interactions primarily acting as module hubs (i.e., 11 in moss, 23 in soil, and 8 in tree hole) (Fig. 4A and B; Table S7). In contrast, in aquatic environments, microeukaryotic specialists played crucial roles within modules as module hubs (i.e., 50 in sediment and 39 in water), while prokaryotic specialists dominated between modules as connectors (i.e., 23 in sediment and 26 in water). Fungi often served as module hubs in five habitats, while many Proteobacteria and Bacteroidota, as well as Metazoa and Chlorophyta, were identified as connectors and module hubs in aquatic environments, respectively (Fig. 4C). Actinobacteriota, as the most abundant keystone taxa, were important in soil networks, demonstrating the distinctive roles of certain taxa within specific habitats (Fig. 4C). Overall, a higher proportion of specialists participated in interdomain interactions as keystone species compared with generalists, especially in aquatic environments (Fig. S10D). After the random removal of half of the generalists or specialists, lower proportions of specialists were retained in the network compared with generalists (Fig. 4D). Additionally, a significantly higher network vulnerability was observed in specialists than generalists (Fig. S11). Altogether, these results highlighted the importance of specialists in maintaining the network stability.

### Evolutionary characteristics of generalist and specialist

The genome lengths of prokaryotic habitat generalists and specialists were evaluated at the family level, and the results showed that generalists had a significantly (*P* < 0.05) larger genome than specialists in most habitats, except moss (Tables S8 and S9). Additionally, for both prokaryotes and microeukaryotes, habitat specialists showed a significantly (*P* < 0.05) longer phylogenetic branch length than generalists (Fig. S12).

**Fig 5:**
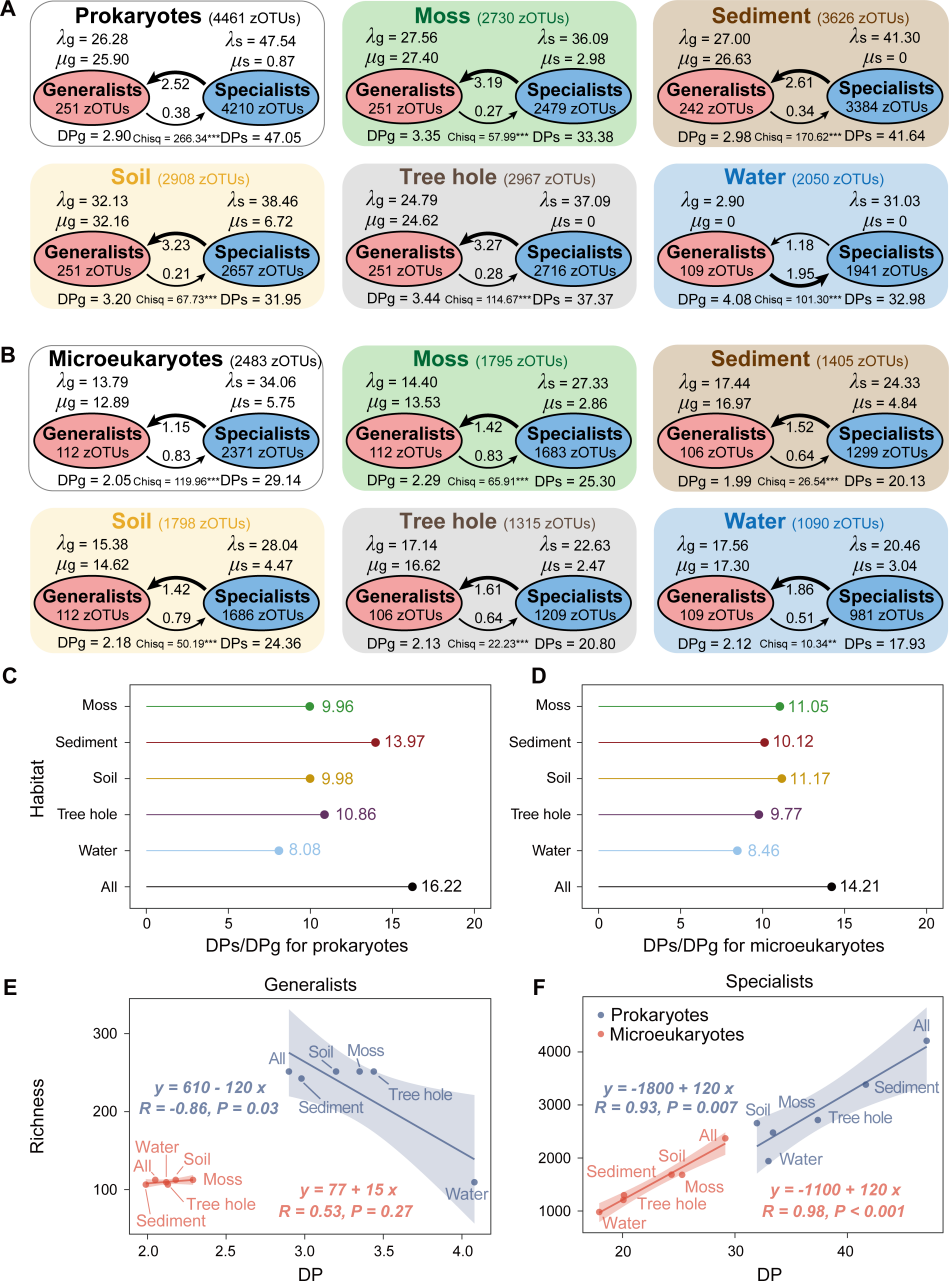
Estimation of the evolutionary characteristics of strict habitat generalists and specialists of prokaryotes and microeukaryotes based on binary-state speciation and extinction (BiSSE) model in five urban park habitats, respectively. (**A, B**) Speciation rates (λg for generalists, λs for specialists), extinction rates (μg for generalists, μs for specialists), state-transition rates between generalists and specialists (arrows), and diversification potential (DPg for generalists, DPs for specialists). A higher value represents a higher rate. Significant level at *P < 0.05, **P < 0.01, and ***P < 0.001 by χ^2^ test. The ratio of DPs to DPg for (**C**) prokaryotes and (**D**) microeukaryotes. Relationships between DP and community richness for prokaryotic and microeukaryotic (**E**) generalists and (**F**) specialists based on the linear model. *R* value is Pearson’s correlation coefficient, and *P* value is the significance level. Shadow indicates 95% confidence interval.

The evolutionary characteristics of habitat generalists and specialists, including species speciation (λ), extinction (μ), and state transition (*t*) rates between strict habitat generalists and specialists, were investigated through the BiSSE model (Fig. 5). Overall, both prokaryotic and microeukaryotic specialists showed higher speciation rates (λs = 47.54 and 34.06) than generalists (λg = 26.28 and 13.79), while specialists displayed considerably lower extinction rates (μs = 0.87 and 5.75) compared with generalists (μg = 25.90 and 12.89) (Fig. 5A and B). Similar patterns were observed across various habitats, with generalists showing comparable speciation and extinction rates, while specialists exhibited a much higher speciation rate than extinction rate. Furthermore, we estimated the diversification potential (DP, DP = λ *+ t −* μ) of generalists and specialists representing the generation rate of biodiversity considering speciation and transition rates (positive effect) as well as extinction rate (negative effect) on microbial diversity. Notably, we found a remarkably higher DP for habitat specialists compared with generalists (Fig. 5C and D), and the ratio of DP for specialists to generalists (DPs/DPg) was slightly higher for prokaryotes (16.22) than for microeukaryotes (14.21). Sediment (13.97) and soil (11.17) displayed the highest DPs/DPg for prokaryotes and microeukaryotes, respectively, while water consistently showed the lowest for both communities. We further linked DP with α-diversity, such as community richness. Among generalists, microbial richness showed a decreasing trend with DP for prokaryotes, mainly driven by water samples, whereas a contrasting trend was identified for microeukaryotes (Fig. 5E and F). There was a significant (*P* < 0.05) positive correlation between DP and richness for both prokaryotic and microeukaryotic habitat specialists (Fig. 5E and F).

## DISCUSSION

### Diversity of habitat generalists and specialists in urban park ecosystems

In our definition, we determined a habitat generalist (or specialist) based on three indices, namely, Levins’ niche breadth, Shannon diversity, and occurrence. Microbial communities consisted of a large number of specialists and a few generalists (Fig. 1; Fig. S3). Consistently, using the same identification method, a few habitat generalists and a significantly large number of specialists were detected in urban aquatic ecosystems (5, 13). Our results clearly demonstrated that the diversity and relative abundance of habitat generalists and specialists were significantly different between the five habitats (Fig. 1), and the habitat type was more important than geographical location in shaping both prokaryotic and microeukaryotic community compositions (Fig. S5). The numbers and relative abundances of specialists in aquatic habitats (i.e., water and sediment) were significantly higher than those in terrestrial habitats (i.e., moss, soil, and tree hole), whereas the opposite trend was observed for generalists, indicating that substantial specialists exclusively thrive in aquatic environments. Habitat specialists and generalists showed distinct community compositions and biogeographical patterns, and specialists varied more noticeably between habitats (environment) or parks (distance) at both taxonomic and phylogenetic levels by Bray-Curtis and UniFrac distances (Fig. S6 to S9), aligning with prior microbial researches on urban aquatic ecosystems as well as natural lakes and ponds (5, 11, 19, 20). We observed that prokaryotes were more diverse than microeukaryotes, and both communities were shown to exhibit high spatial turnover rates in different local habitats (Fig. S8 and S9). These results collectively demonstrate spatial niche differentiation in community composition among different microbial types (i.e., prokaryotes and microeukaryotes) and compositions (i.e., habitat specialists and generalists). A recent study in different urban aquatic ecosystems in central China corroborated our results that niche differentiation controls microbial diversity and community composition (5). Furthermore, local ecological conditions in various habitats regulate microbial diversity and community composition (11, 19).

### Microbial diversity and community assembly regulated by different ecological processes

Until now, a comprehensive study of cross-kingdom prokaryotes and microeukaryotes together in multiple habitats in urban park ecosystems and their underlying ecological processes has not been well documented. Our results showed that stochastic processes consistently dominated community assembly in both prokaryotes and microeukaryotes in urban parks (Fig. 2). Importantly, dispersal limitation and homogeneous selection played distinct roles in shaping the community assembly, especially as these processes were stronger for habitat specialists compared with generalists (Fig. 2), indicating geographical distance and environmental filtering jointly regulated the diversity and distribution of microbial communities, especially for microbial specialists. Moreover, local environmental condition, such as anthropogenically polluted urban water and sediment (5, 13), changing salinity and nutrients (11), and regional environmental conditions such as terrestrial-freshwater gradient (21), might create strong environmental partition to the community assembly, leading to strong deterministic processes of selection. Consistent with previous findings, our results revealed that the relative importance of deterministic processes was stronger for habitat specialists than generalists in most habitats, indicating habitat specialists were more susceptible to environmental changes than generalists. Therefore, when confronted with diverse natural environmental changes or anthropogenic activity disturbances, specialists might be more prone to making significant alterations in the ecosystems than generalists. These changes, in turn, can profoundly impact the health and functionality of urban parks. Understanding the mechanisms behind these ecological processes is important for taking proper urban ecosystem management initiatives.

### Microbial interaction network strongly regulated by habitat specialists

In this study, a significant number of positive intradomain and interdomain interactions were primarily observed compared with negative ones (Fig. 3; Tables S5 and S6), indicating mutualistic relationships among the microbial communities and these positive associations are key in maintaining biodiversity and ecosystem functions (22). We further observed a significantly higher number and proportion of habitat specialists compared with generalists in aquatic networks (Fig. 3), which is consistent with previous findings in both natural and urban aquatic ecosystems (5, 14). Most importantly, higher proportions of specialists were also identified as keystone taxa than the generalists in microbial network (Fig. 3; Fig. S10B and D), suggesting microbial specialists are disproportionately important in maintaining ecosystem stability and functioning (4, 14). Additionally, a higher microbial diversity was observed in soil compared with other habitats (23), while in our study, a higher percentage of generalists participated in biological interactions in soil, and a higher proportion of specialists was found in other habitats (Fig. S10A and C), implying that generalists were more involved in interactions in soil habitats, whereas specialists maintained biological interactions in the other habitats. The larger genome size of generalists might make them less dependent on the presence of other microbes for survival (Table S9). However, specialists with a smaller genome size are expected to rely more on external metabolites or nutrients generated by other microbes for growth; therefore specialists may dominate biological interactions and serve more as keystone species than generalists.

Notably, generalists and specialists displayed significantly different ecological roles in intradomain and interdomain interactions, where many more specialists interplay with other species as module hubs than connectors (Fig. 3 and 4), indicating their central role in connecting nodes within module (14, 24). In cross-kingdom interactions, prokaryotic habitat specialists acted in different ways, mainly as module hubs in the terrestrial ecosystems, while connectors in the water environment (e.g., Proteobacteria and Bacteroidota) (Fig. 4). Concurrently, microeukaryotes (e.g., Metazoa, Fungi, and Chlorophyta) became active as module hubs to maintain the stability of the aquatic community. Thus, these results suggested that a species (whether habitat generalists or specialists) could act differently (e.g., module hubs or connectors) to maintain hierarchical interaction patterns regarding habitat types, to ensure stable microbial interactions and to maintain the community structure. Hence, results from our analysis provide new insights into the complex diversity of interactions in urban park ecosystems. Determining the specific ecological roles and functions of these key species within a specific habitat will be a significant research focus. This insight can inform the development of more targeted and sustainable strategies for protecting biodiversity and enhancing the resilience of urban parks.

### Microbial biodiversity regulated by evolutionary processes

Speciation is the ultimate driver of diversity (8). We found prokaryotes had a faster speciation rate but a lower extinction rate than microeukaryotes, which suggested a higher diversification potential for prokaryotes (Fig. 5). Furthermore, a strong positive correlation between diversification potential and richness for habitat specialists demonstrated that evolutionary processes were important for generating, adapting, and maintaining of microbial diversity, especially for prokaryotes. Importantly, several lines of evidence estimate that generalists with broader niche breadth tend to become specialists with narrow niche breadth in urban park ecosystems, whereas the opposite process is more challenging. Firstly, we found that habitat specialists had a much higher diversification potential than generalists from the BISSE model (Fig. 5). A previous study revealed that broad niche and high dispersal abilities had strong negative effects on speciation rates (25). Consistently, habitat generalists showed a relatively low speciation rate as well as a comparable extinction rate. Secondly, the phylogenetic branches for habitat generalists were significantly shorter than those of specialists (Fig. S12), suggesting that the currently existing habitat generalists have a relatively short evolutionary period from the ancestor and are likely to grow earlier than specialists in urban parks. Thus, its descendants could spread into new environments, where they can be colonized and specialized (10), while habitat specialists with a high diversity may occur later under persistent environmental filtering. Thirdly, our findings indicated that prokaryotic habitat generalists tended to possess larger genomes across various urban park habitats (i.e., soil, sediment, tree hole, and water) (Table S9), which may better support their survival under changing environmental conditions (10). According to the black queen hypothesis (26), generalists are able to reduce their genome size in a suitable environment, thereby reducing metabolic consumption and becoming a local ecotype as well as acting as specialists. Overall, this potential transition tendency from habitat generalists to specialists in urban parks has in turn been able to change environmental conditions and affect ecosystem functioning. Importantly, it has been proposed to be further quantified as an index to assess environmental changes (such as climate and habitat disturbances) on microbial biodiversity (27).

Habitat specialists occupy a narrow ecological niche and are subject to strong environmental filtering. Additionally, evolutionary processes such as genetic constraints and trade-offs collectively render them vulnerable to environmental changes, local extinctions, and range fragmentation (18). Consequently, they might undergo more frequent allopatric speciation, leading to greater diversification compared with generalists. It should be noted that habitat generalists showed higher speciation and lower extinction rates than specialists in some studies (10, 15, 18). This inconsistency may come from our definition of habitat generalists and specialists that combines three kinds of niche breadth indices (i.e., Levins’ niche breadth, Shannon diversity, and occurrence), while classification in other studies was often based on species occurrence in specific environment groups independent of the relative abundance. It is important to mention that different classification methods may vary greatly in retained habitat generalists and specialists and further affect evolutionary characteristics as well. Moreover, different spatial scales indicate discrepant species pools, which can also have a great influence on the results. Furthermore, the acceleration of urbanization can precipitate abrupt ecological shifts within urban areas. These shifts have the potential to generate novel ecological niches and to modulate species turnover rates. As a result, it may trigger distinct evolutionary and ecological processes when compared to those observed in natural ecosystems. In summary, our research emphasizes the importance of habitat specialists in the interaction networks and species diversification, along with their vulnerability to environmental changes, highlighting their significance for conservation efforts and ecosystem management.

### Conclusions

**Fig 6:**
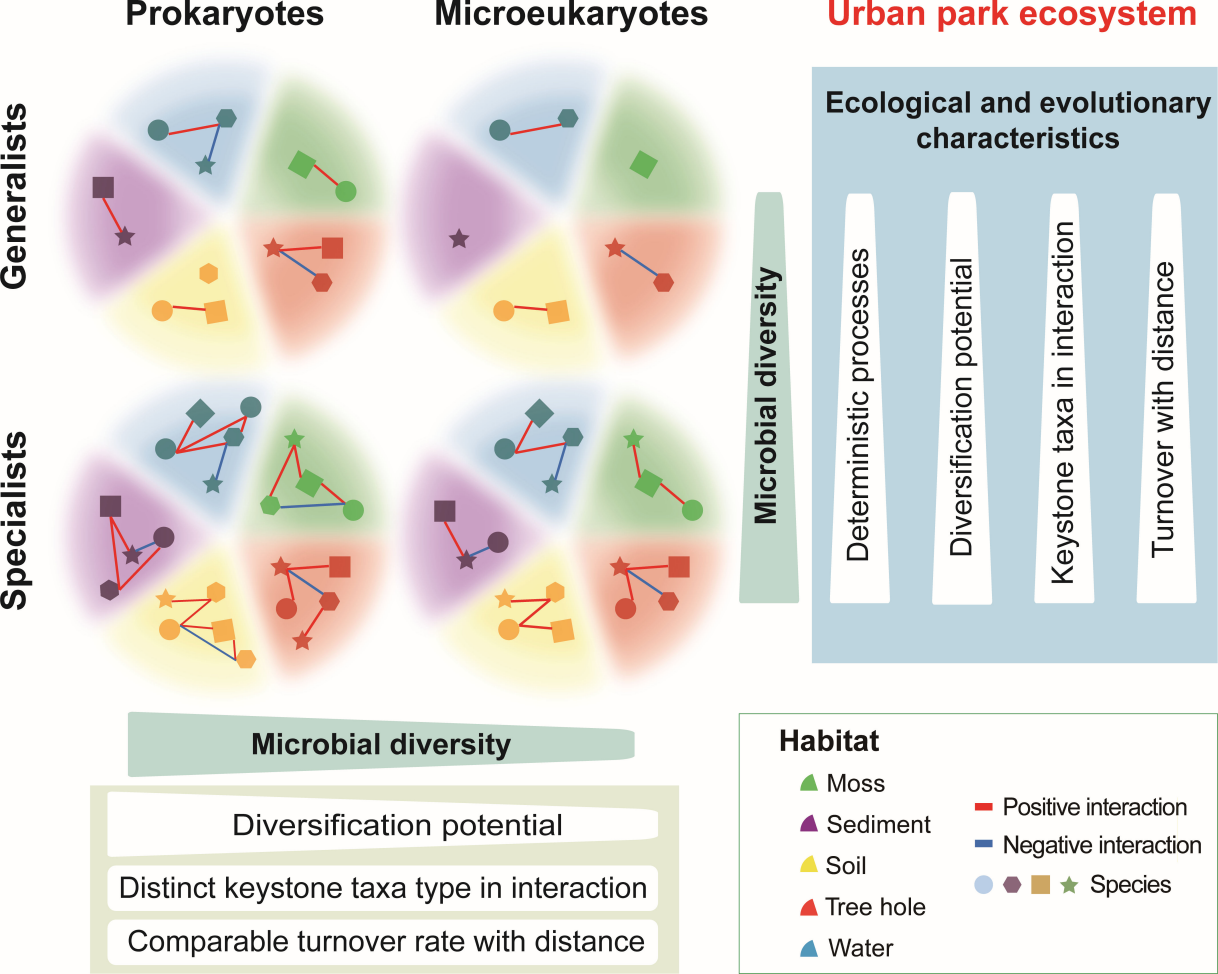
A conceptual framework explaining the ecological and evolutionary characteristics of prokaryotes and microeukaryotes in urban microbiomes.

To the best of our knowledge, this is the first study exploring the diversity patterns of prokaryotic and microeukaryotic habitat generalists and specialists in urban park ecosystems based on a representative set of different habitats. We found that both ecological and evolutionary drivers have a major impact on microbial diversity in urban park microbiomes (Fig. 6). Stochastic processes dominated the community assembly of prokaryotic and microeukaryotic habitat generalists and specialists. Deterministic processes were found important in shaping microbial communities, especially habitat specialists, but their influence varied and depended on the habitat type. To be specific, prokaryotes, particularly habitat specialists, exhibited higher diversification potentials and diversity compared with microeukaryotes and habitat generalists, respectively. Furthermore, a distinct role (e.g., module hubs or connectors) of prokaryotic and microeukaryotic habitat specialists and generalists in hierarchical interaction maintained differentiated community diversity, network stability, and community interactions. The geographical distance and environmental filtering jointly regulated microbial diversity through different community assembly processes and turnover rates. Thus, our findings highlighted the role of habitat ecological and evolutionary features in shaping the microbial diversity of prokaryotic and microeukaryotic generalists and specialists in urban park ecosystems across multi-habitat. As rapid urbanization has been shown to reduce microbial diversity, notably, habitat specialists have been more vulnerable to extinction than generalists. Furthermore, the transition from habitat generalists to specialists indicates that the microbial niche breadth may shrink due to changing urban environments. The findings of the study would suggest that more research is necessary in the future, especially focusing on time series data from urban ecosystems, to demonstrate the dynamics of the speciation, extinction, and turnover of habitat generalists and specialists, in order to a better prediction of their effects on the environment and ecosystem services.

## MATERIALS AND METHODS

### Sample collection and sequencing

Xiamen City (24.48 N, 118.08 E), in Fujian province, China, is characterized by a subtropical monsoon climate. A total of 90 samples were collected in July 2020 in six urban parks (Fig. S1). In each park, samples were collected from five habitats, including mosses (100 g), surface soils up to a depth of 5 cm (100 g), litter or detritus in tree holes (100 g), surface pond sediments (100 g), and surface water to a depth of 0.5 m (2.5 L), and processed following the previously described methods (23). Then, total DNA was extracted, and polymerase chain reaction assays were performed targeting the V3–V4 region of the 16S rRNA gene and the V4 region of 18S rRNA gene using universal primer pairs, specifically 341F/806R (28) and 547F/967R (29), respectively. Three replicated PCR products were mixed, purified, and sequenced on an Illumina platform (Illumina Inc., San Diego, CA, USA).

### Sequence processing

Paired-end high-throughput sequencing reads were filtered for quality control using QIIME V1.9.1 (30). Chimeric sequences were discarded using the UCHIME algorithm (31). Subsequently, the UNOISE3 algorithm was applied to generate zOTUs (32). zOTUs with fewer than 10 sequences were removed to minimize sequencing errors (23). Finally, zOTU tables were rarefied to a minimum number of sequences from each sample, at 48,116 for prokaryotes and 86,235 for microeukaryotes, to normalize sequencing effects (33). The total data set contained 42,618 and 15,112 zOTUs for prokaryotes and microeukaryotes in all samples, respectively. Representative sequences were assigned to taxonomic lineages using the SILVA database (v138.1) for prokaryotes (34) and PR^2^ database (v4.14.0) for microeukaryotes (35).

### Identification of habitat generalists, specialists, and opportunists

It should be noted that the definition of habitat generalists and specialists is normally based on the niche theory; however, the specific methods employed can lead to variations in the observed abundances of habitat generalists and specialists across studies. The identification of habitat generalists and specialists can be established through various methods, including Levins’ niche breadth index (11, 19), species occurrence in several environment clusters/types (10, 18), the coefficient of variation of taxon abundances across samples (36), or organisms occurring in compositionally similar or diverse samples (37). In this study, to determine the habitat generalists and specialists more preciously, three niche breadth-related indices, including Levins’ niche breadth, Shannon diversity, and occurrence frequency of zOTUs were all calculated using the “EcolUtils” package (38). To facilitate comparison between habitats, we considered various habitats in urban parks as a unified ecosystem, identifying habitat generalists and specialists and assigning them to each habitat. Each zOTU was classified as a habitat generalist or specialist based on whether the observed values were higher or lower than the 95% confidence interval for all three indices (5). This method used a null model computed by permutating community abundance tables to generate a series of random distributions; then, the observed value was compared to assess if it significantly deviated from this distribution. Therefore, this method can minimize possible biases introduced by a single indexing method, such as relying on only one index or subjectively selecting cutoffs for defining habitat generalists and specialists (11, 18, 19). Furthermore, to reduce the complexity and potential noise in the data sets, we designated the generalists from the upper 10th percentile of all three-index values as strict habitat generalists (39). Since rare taxa may be below detection limits or temporarily absent in some habitats, they could potentially be incorrectly identified as specialists. Hence, we only considered specialists with over 50 reads and defined them as strict habitat specialists following previous studies (13, 14). Only these strict habitat generalists and specialists were retained for subsequent analysis. Other species being neither strict habitat generalists nor specialists were marked as opportunists. The assignment of habitat generalists and specialists of prokaryotic and microeukaryotic zOTUs, their abundances, classifications, and the three niche breadth values are presented in Tables S1 and S2.

### Statistical analysis

Rarefaction curves and Good’s coverage were obtained to explore whether the sequencing depth was enough for community analysis. The community composition variation and the relative contribution of habitat and park were explored by hierarchical cluster analysis based on Bray-Curtis dissimilarity using the “ward.D2” method in R (40); furthermore, the clustering features were visualized by the “ggtree” package (41). PERMANOVA was performed to show whether community composition variation was significant across the five habitat types using the “adonis2” function in R. Additionally, a non-metric multidimensional scaling (NMDS) analysis based on the Bray-Curtis dissimilarity matrix, as well as a principal co-ordinates analysis (PCoA) based on weighted UniFrac dissimilarity, was applied to visualize community composition variation with ANOSIM (analysis of similarity tests) using the “vegan” (42), “picante” (43), and “ape” (44) packages.

We investigated the relationships between community dissimilarity and geographical distance (distance-decay relationships) of habitat generalists and specialists based on the taxonomic and phylogenetic dissimilarity matrix (45), using the following equation:


Log (dissimilarity)=β×log (D+1)+c

Where dissimilarity is the community dissimilarity, β is the slope (i.e., spatial turnover rate). *D* is the geographical distance (Euclidean distance), and *c* is the intercept parameter (one is added to *D* before taking the logarithm to avoid negative infinity).

### Community assembly mechanisms

The relative importance of stochastic and deterministic processes in the community assembly of prokaryotic and microeukaryotic habitat generalists and specialists was quantified using the phylogenetic-bin-based null model with the “iCAMP” package (46). Within this framework, ecological processes can be categorized into homogeneous selection and heterogeneous selection (i.e., deterministic processes), as well as homogenizing dispersal, dispersal limitation, and drift (i.e., stochastic processes) (47). The processes are first identified based on phylogenetic diversity null model analysis using the beta nearest-taxon index (βNTI). If βNTI < − 2 and βNTI > 2, the community assembly is driven by homogeneous selection and heterogeneous selection, respectively. Furthermore, the taxonomic diversity matrix based on the modified Raup-Crick (RC) index is applied to summarize stochastic processes. If |βNTI| < 2 and RC < −0.95 and >0.95, the community assembly is driven by homogenizing dispersal and dispersal limitation, respectively. The remains represent drift, diversification, weak selection, or weak dispersal and are simply denoted as drift.

### Construction of intradomain and interdomain ecological networks

Ecological interactions can be represented by ecological networks. We used FastSpar to implement the SparCC algorithm for inferring microbial correlation networks (48). Only strong and significant correlations (|*R*| ≥ 0.6; *P* < 0.05) were retained and visualized in Gephi 0.9. Modules were defined by using the greedy modularity optimization (16). To disentangle the role of habitat generalists and specialists in overall interspecific interactions, zOTUs with more than 50 reads were retained to construct intradomain networks within each habitat for prokaryotes and microeukaryotes, respectively. Furthermore, to investigate the potential interactions between habitat generalists and specialists, the opportunists zOTUs were discarded and only habitat generalists and specialists were used to construct interdomain networks and illustrate cross-trophic interactions between prokaryotes and microeukaryotes.

Based on within-module connectivity (*Zi*) and among-module connectivity (*Pi*), zOTUs were classified into network hubs (*Zi* ≥ 2.5; *Pi* ≥ 0.62), module hubs (*Zi* ≥ 2.5; *Pi* < 0.62), connectors (*Zi* < 2.5; *Pi* ≥ 0.62), and peripherals (*Zi* < 2.5; *Pi* < 0.62) (49). Network properties were calculated based on a pipeline (50), including *R*^2^ of power-law, average clustering coefficient (avgCC), average path distance (GD), and modularity for intradomain networks. For interdomain networks, niche overlap (similarity of the interaction pattern between species), robustness (the tolerance of the system to species extinctions), and functional complementarity (the total branch length of a functional dendrogram based on differences in visitor assemblages) were calculated (17). Specifically, we randomly removed 50% of the habitat generalist or specialist zOTUs in each network, and the remaining proportion of zOTUs in the network was calculated to compare the role of habitat generalists and specialists in network stability. Vulnerability was measured according to the contribution of the network node to the global efficiency (51). Additionally, randomized networks were made by rewiring empirical network results 100 times.

### Phylogenetic branch length, prokaryotic genome size, and binary-state speciation and extinction model

To investigate evolutionary features, microbial phylogenetic branch length and prokaryotic genome size were estimated, and the speciation and extinction rates of habitat generalists and specialists were calculated. The representative sequences of zOTUs were utilized to construct a phylogenetic tree and calculate phylogenetic distances. Specifically, phylogenetic distance (the cophenetic distance) within habitat generalists or specialists in each habitat was calculated after rooting the phylogenetic tree using the “pdist.big” function in the “iCAMP” package (46, 52). The phylogenetic branch length was then calculated based on the phylogenetic distance between the root node and each habitat generalist or specialist. We used the Pathosystems Resource Integration Center (PATRIC) genome database (37) which contains 225,101 high-quality prokaryotic genomes representing 34,304 species as the reference for mapping the taxonomic information of zOTUs. As a result, about half of zOTUs could be aligned at the class, order, and family levels (Table S8). Considering both accuracy and specificity, we explored whether prokaryotic habitat generalists and specialists exhibit significant differences in genome size at the family level.

The binary-state speciation and extinction (BiSSE) model was applied to estimate species speciation (λ), extinction (μ), and state transition (*t*) rates between extant strict habitat generalists and specialists (53). Briefly, this model comprises two steps. Step 1 assumes a starting point for the simulation using the “starting.point.bisse” function. At this point, the speciation and extinction rates of habitat generalists and specialists were constrained the same. Step 2 allows all rates to be different and applies maximum likelihood method to calculate the evolutionary rate parameters through the “find.mle” function. Analysis of variance was applied to test whether the results significantly differed between these two-round runs. The BiSSE model analysis was implemented in the “diversitree” package (54). Additionally, considering the positive contribution of speciation and transition rates and the negative contribution of the extinction rate to microbial diversity, the DP was used to characterize the generation rate of diversity based on a previous study (8) using the following equation:


DP=λ+t−μ

where DP indicates microbial diversification potential, λ indicates the speciation rate, *t* indicates the transition rate, and µ indicates the extinction rate. Therefore, DP is derived from existing species and can reflect community diversity dynamics over future time periods.

## Data Availability

All sequencing data have been uploaded to the public NCBI Sequence Read Archive (SRA) (16S rRNA gene V3–V4 region, PRJNA752455; 18S rRNA gene V4 region, PRJNA757135) and NODE database (https://www.biosino.org/) (16S rRNA gene V3–V4 region, OEP001968; 18S rRNA gene V4 region, OEP002253).

## SUPPLEMENTAL MATERIAL

Supplemental material msystems.00469-24-s0001.pdf Fig. S1-S12; table captions; Tables S3-S6, S8, and S9.

Table S1 msystems.00469-24-s0002.csv Prokaryotic zOTUs.

Table S2 msystems.00469-24-s0003.csv Microeukaryotic zOTUs.

Table S7 msystems.00469-24-s0004.xlsx Identified keystone species in interdomain networks and their taxonomic annotations.

ASM does not own the copyrights to Supplemental Material that may be linked to, or accessed through, an article. The authors have granted ASM a non-exclusive, world-wide license to publish the Supplemental Material files. Please contact the corresponding author directly for reuse.
