# Supplementary material for: Ecological and evolutionary processes involved in shaping microbial habitat generalists and specialists in urban park ecosystems
Source: mSystems. 2024 May 20;9(6):e00469-24. doi: 10.1128/msystems.00469-24 (PMC11237591; doi:10.1128/msystems.00469-24)
Supplement: Supplemental material — Fig. S1-S12; table captions; Tables S3-S6, S8, and S9. [file msystems.00469-24-s0001.pdf]

Supplementary information

Ecological and evolutionary processes involved in shaping microbial habitat generalists and specialists in urban park ecosystems

Shuzhen Li,[1,*] Xue Yan,[1,2,*] Mamun Abdullah Al,[1] Kexin Ren,[1] Christopher Rensing,[1,3] Anyi Hu,[4] Andrey N. Tsyganov,[5] Yuri Mazei,[5,6,7] Alexey Smirnov,[8] Natalia Mazei,[5] Jun Yang,[1]#

[1] *Aquatic EcoHealth Group, Fujian Key Laboratory of Watershed Ecology, Key Laboratory of Urban Environment and Health, Institute of Urban Environment, Chinese Academy of Sciences, Xiamen 361021, China*

[2] *University of Chinese Academy of Sciences, Beijing 100049, China*

[3] *Institute of Environmental Microbiology, College of Resources and the Environment, Fujian Agriculture & Forestry University, Fuzhou 350002, China*

[4] *CAS Key Laboratory of Urban Pollutant Conversion, Institute of Urban Environment, Chinese Academy of Sciences, Xiamen 361021, China*

[5] *Lomonosov Moscow State University, Leninskiye Gory 1, Moscow 119991, Russia*

[6] *Faculty of Biology, Shenzhen MSU-BIT University, Shenzhen 518172, China*

[7] *A.N. Severtsov Institute of Ecology and Evolution, Russian Academy of Sciences, Leninskiy Ave. 33, Moscow 117071, Russia*

[8] *Department of Invertebrate Zoology, Faculty of Biology, St. Petersburg University, St Petersburg 199034, Russia*

Running title:

Microbial ecology and evolution in urban parks

* These authors contributed equally to this study.

Corresponding author:

E-mail: jyang@iue.ac.cn (J. Yang).

This supplementary information contains:

- 21 Pages
- 12 Figures
- 9 Tables

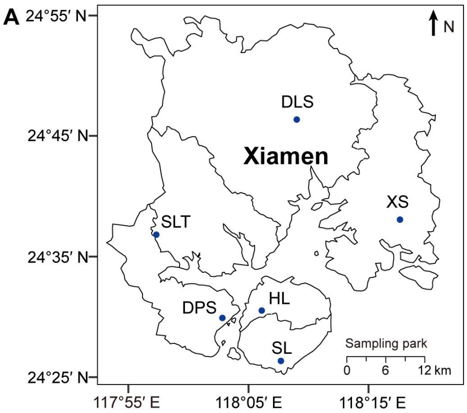

B

Park	ID	Latitude (N)	Longitude (E)
Shuanglongtan	SLT	24.6153°	117.9497°
Dapingshan	DPS	24.5045°	118.0545°
Huli	HL	24.5081°	118.1008°
Shangli	SL	24.4506°	118.1186°
Dalunshan	DLS	24.7467°	118.1475°
Xiangshan	XS	24.6308°	118.2944°

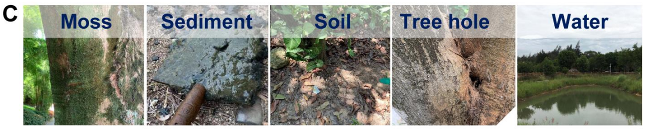

Figure S1 Sampling locations in urban parks in Xiamen city. **(A)** Locations of sampling sites in Xiamen City. **(B)** The coordinates of six parks. **(C)** Pictures of five sampling habitats.

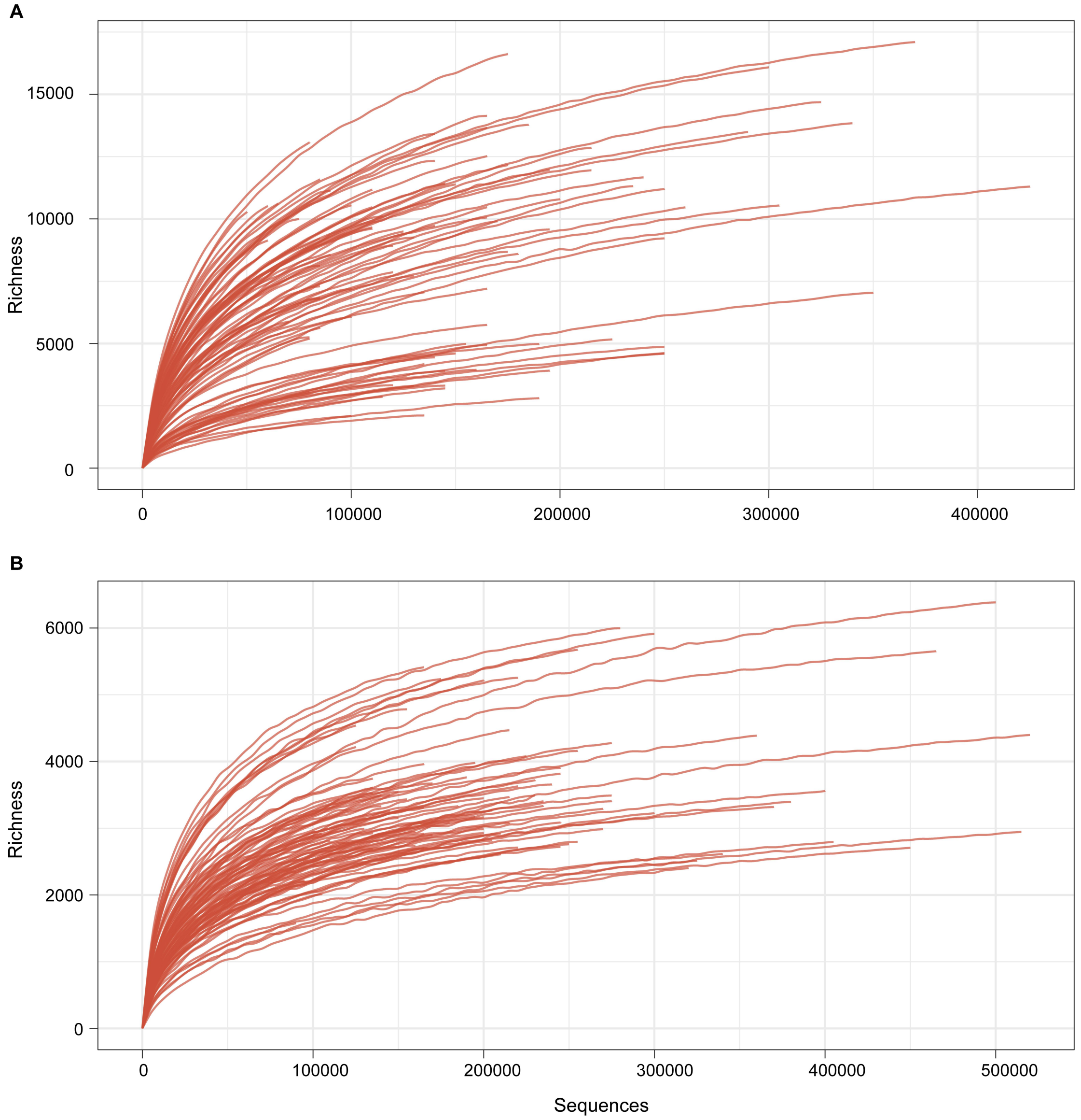

Figure S2 Rarefaction curves of **(A)** prokaryotic and **(B)** microeukaryotic zero-radius operational taxonomic units (zOTUs) from 90 samples.

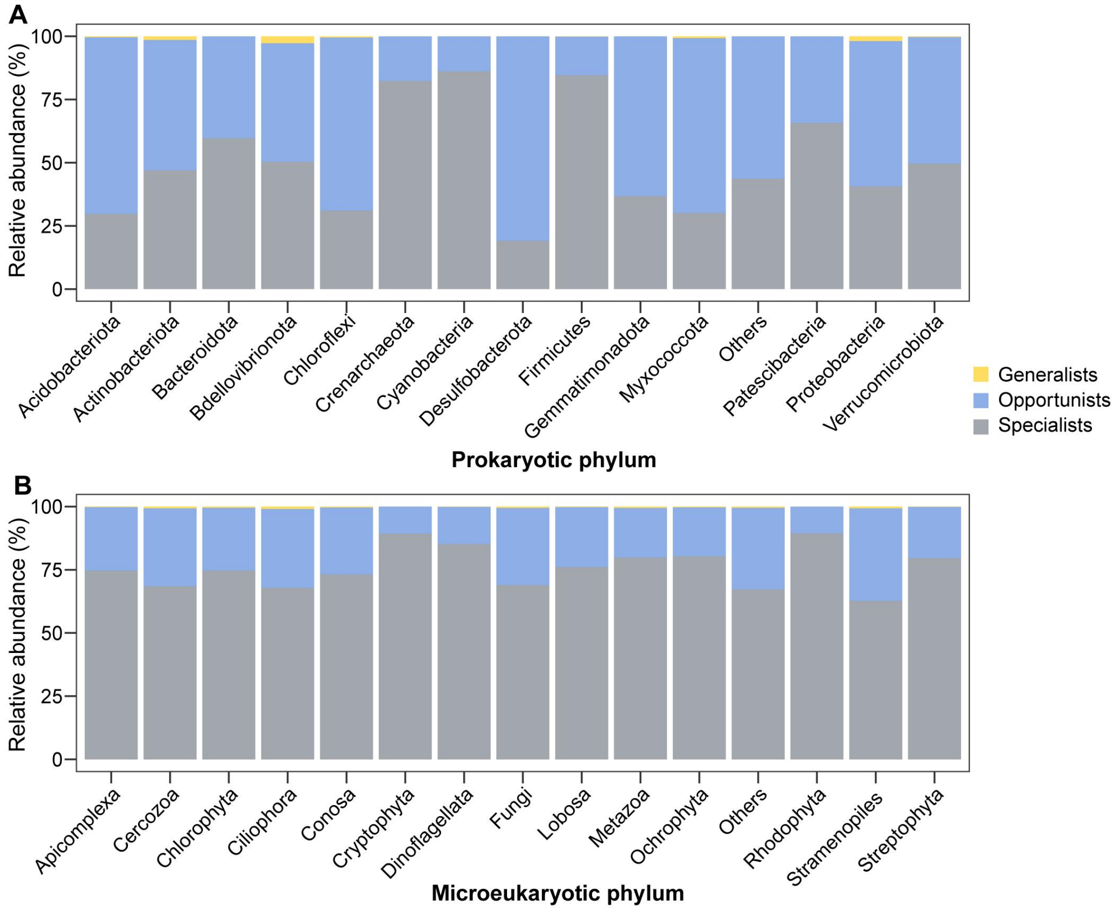

Figure S3 Relative abundance (%) of identified strict generalists and specialists at phylum level of **(A)** prokaryotes and **(B)** microeukaryotes, respectively.

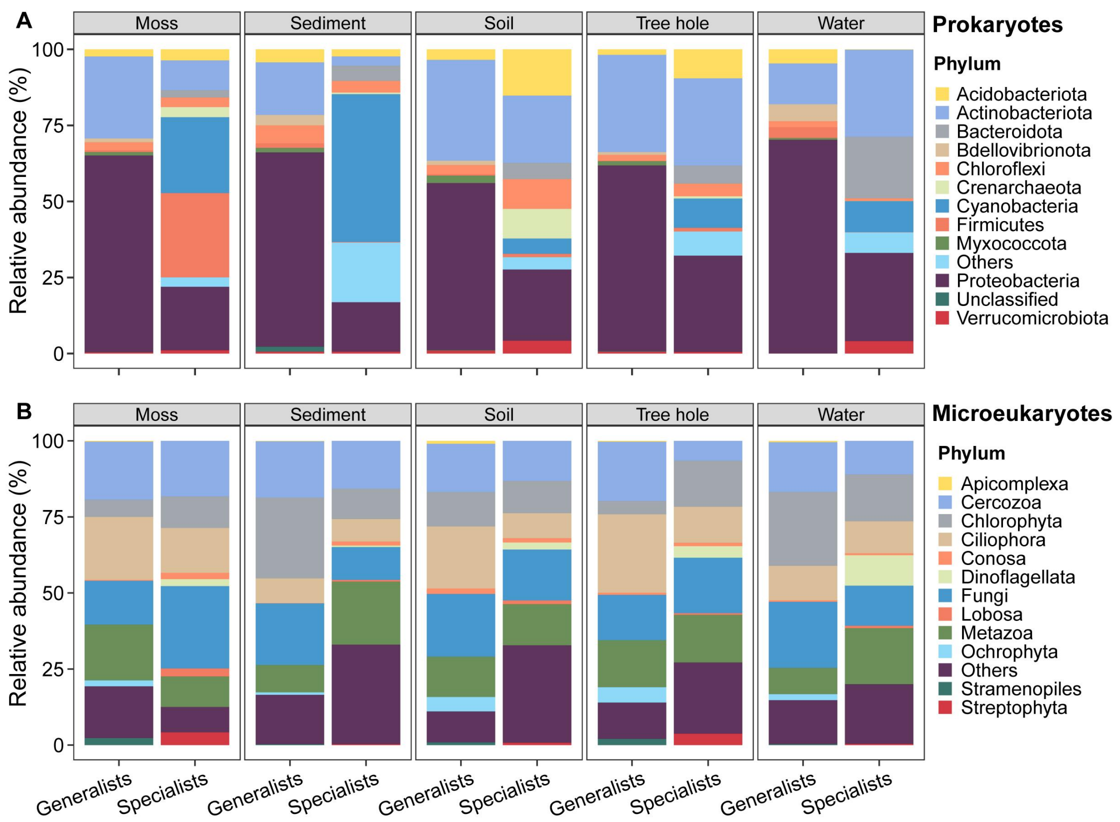

Figure S4 Taxonomic information of habitat generalists and specialists at the phylum level of **(A)** prokaryotes and **(B)** microeukaryotes, respectively. The 12 most abundant phyla are presented. "Others" represents the sum of all other phyla.

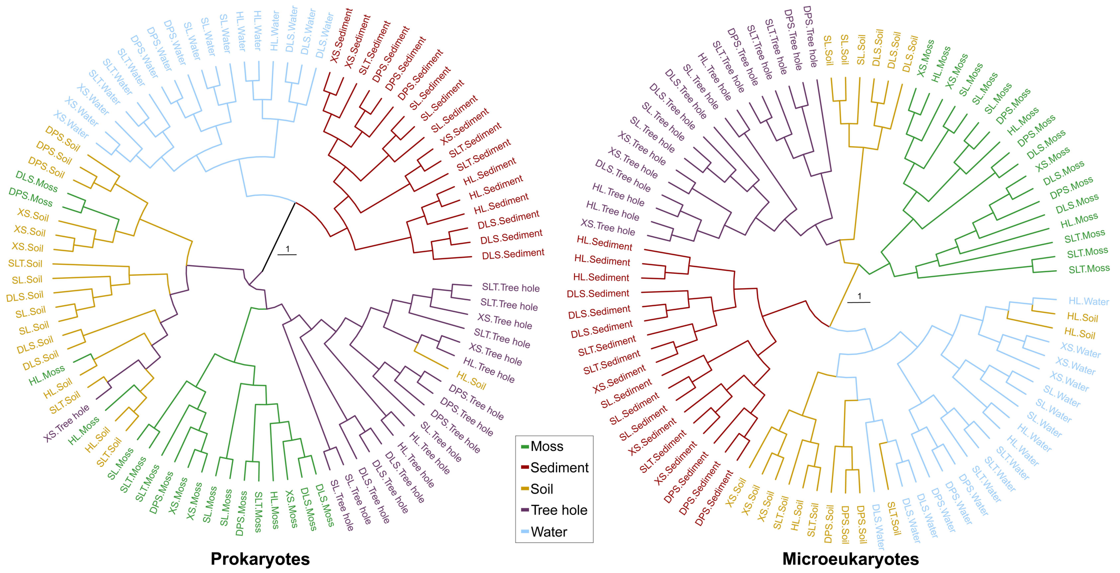

Figure S5 Hierarchical cluster analysis of 90 samples based on the "ward.D2" method from five habitats (i.e., moss, sediment, soil, tree hole, and water), showing the habitat effect on the microbial community. Prokaryotes and microeukaryotes were obtained based on the high-throughput sequencing of 16S rRNA gene V3-V4 region and 18S rRNA gene V4 region, respectively.

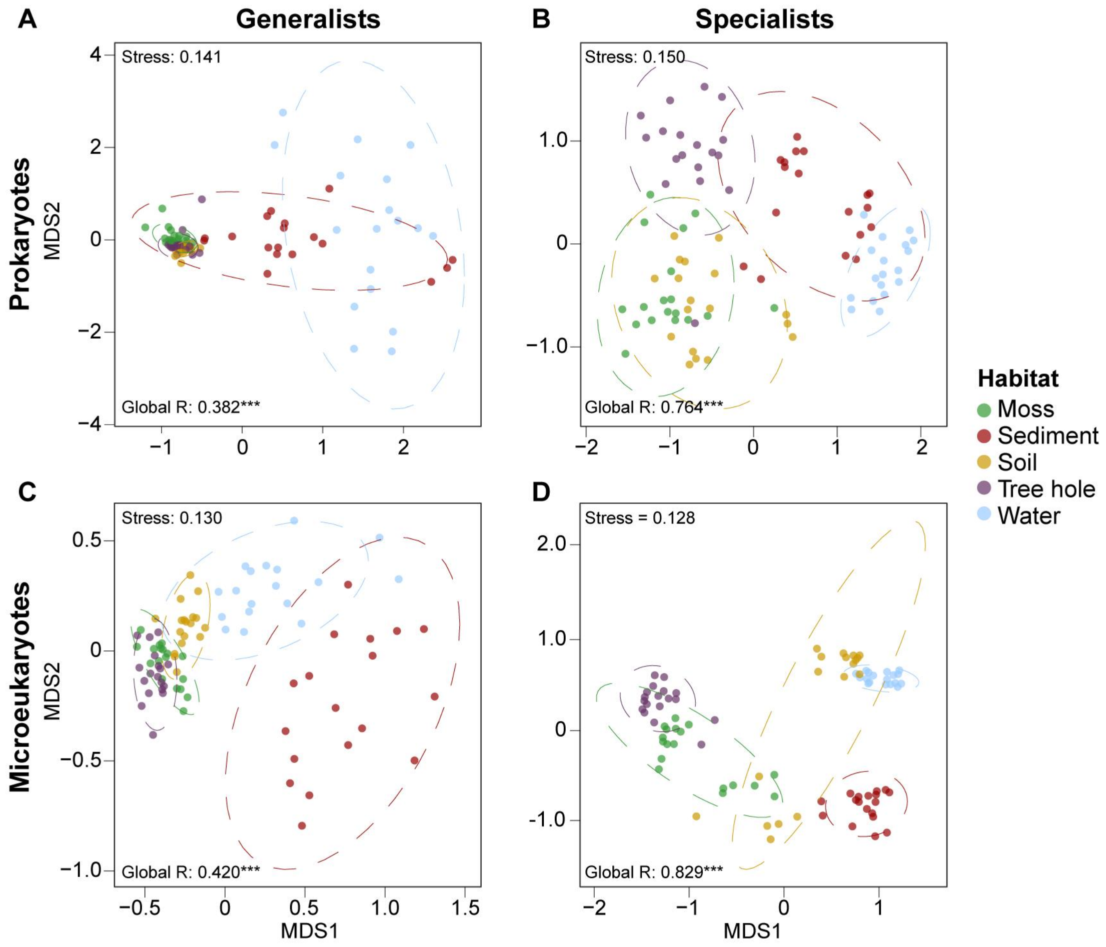

Figure S6 Results of non-metric multidimensional scaling (NMDS) analysis based on Bray-Curtis dissimilarity of prokaryotic habitat (A) generalists and (B) specialists, and microeukaryotic habitat (C) generalists and (D) specialists in urban parks. ***$P <$ 0.001.

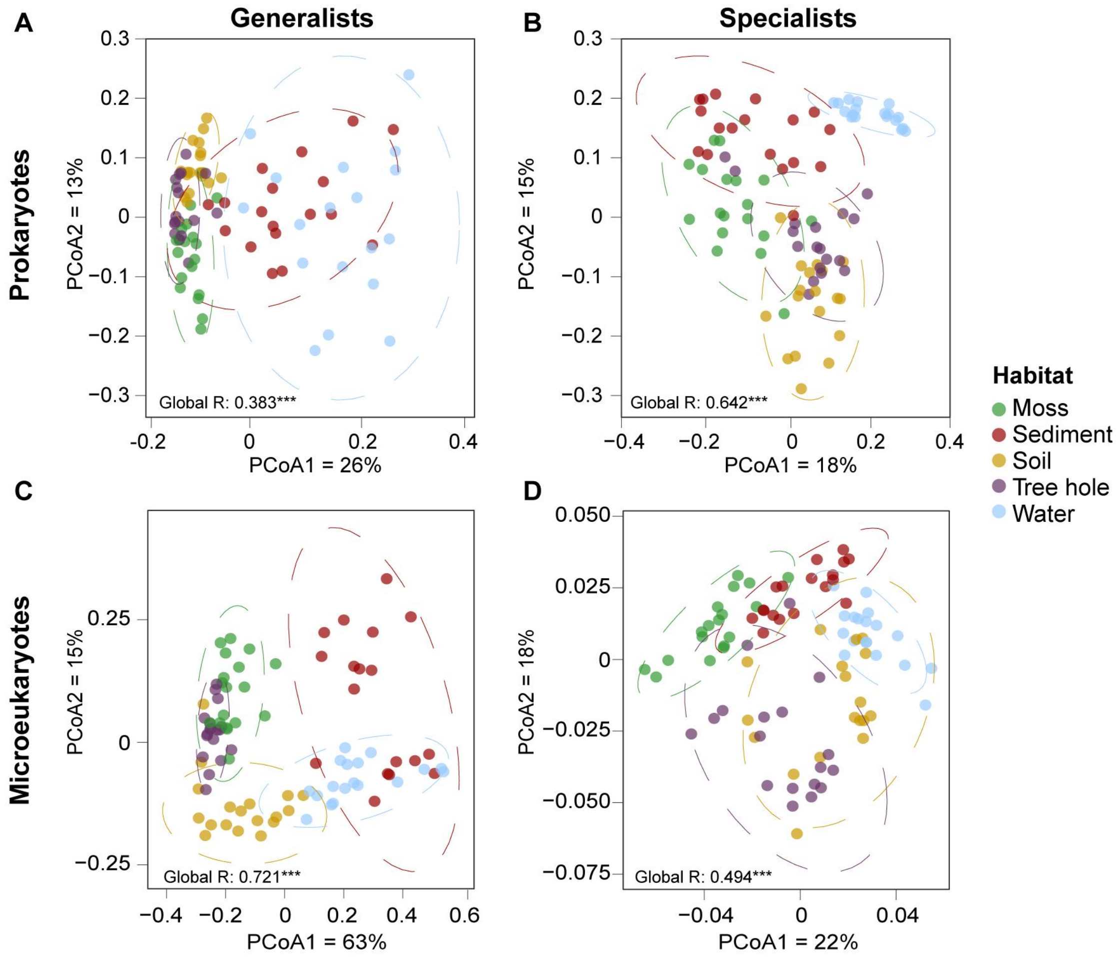

Figure S7 Results of principal co-ordinates analysis (PCoA) based on weighted UniFrac dissimilarity matrix of prokaryotic habitat (A) generalists and (B) specialists, and microeukaryotic habitat (C) generalists and (D) specialists in urban parks. ***$P < 0.001$.

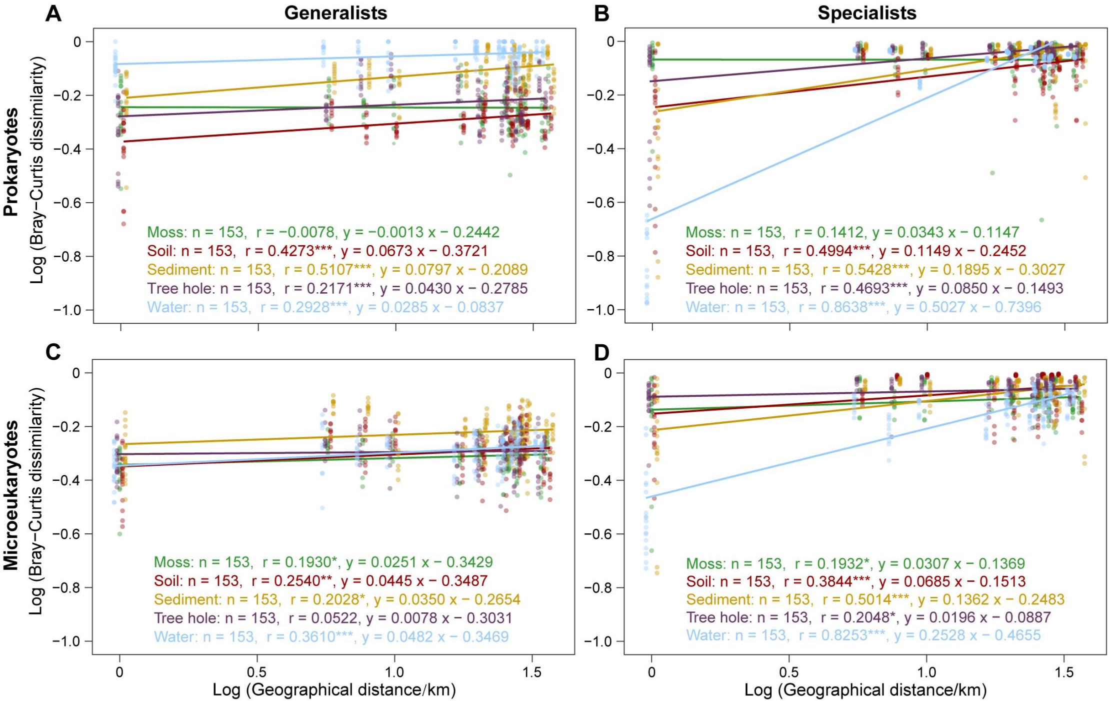

Figure S8 Relationships between Bray-Curtis dissimilarity and spatial distance of prokaryotic habitat **(A)** generalists and **(B)** specialists, as well as microeukaryotic habitat **(C)** generalists and **(D)** specialists in urban parks. A linear regression was fitted between community dissimilarity and spatial distance of pairwise samples. n is the number of sample pairs. *r* value is the Pearson's correlation coefficient, and star represents significance. *$P < 0.05$, **$P < 0.01$, ***$P < 0.001$.

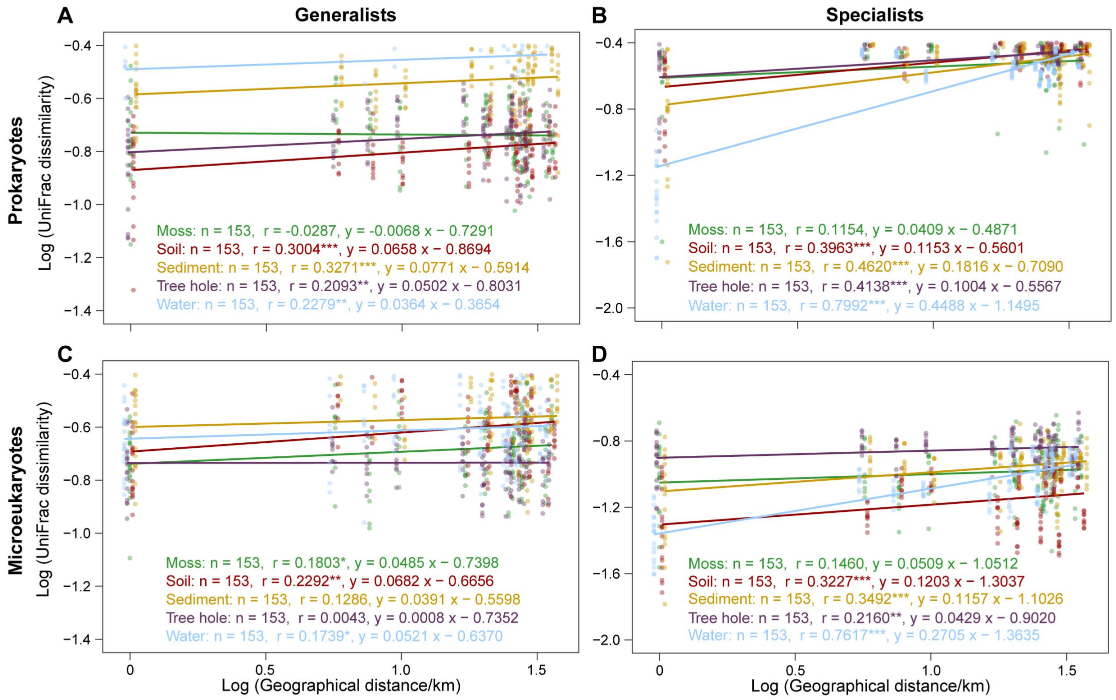

Figure S9 Relationships between weighted UniFrac dissimilarity and spatial distance of prokaryotic habitat **(A)** generalists and **(B)** specialists, as well as microeukaryotic habitat **(C)** generalists and **(D)** specialists in urban parks. A linear regression was fitted between community dissimilarity and spatial distance of pairwise samples. n is the number of sample pairs. *r* value is the Pearson’s correlation coefficient, and star represents significance. *$P < 0.05$, **$P < 0.01$, ***$P < 0.001$.

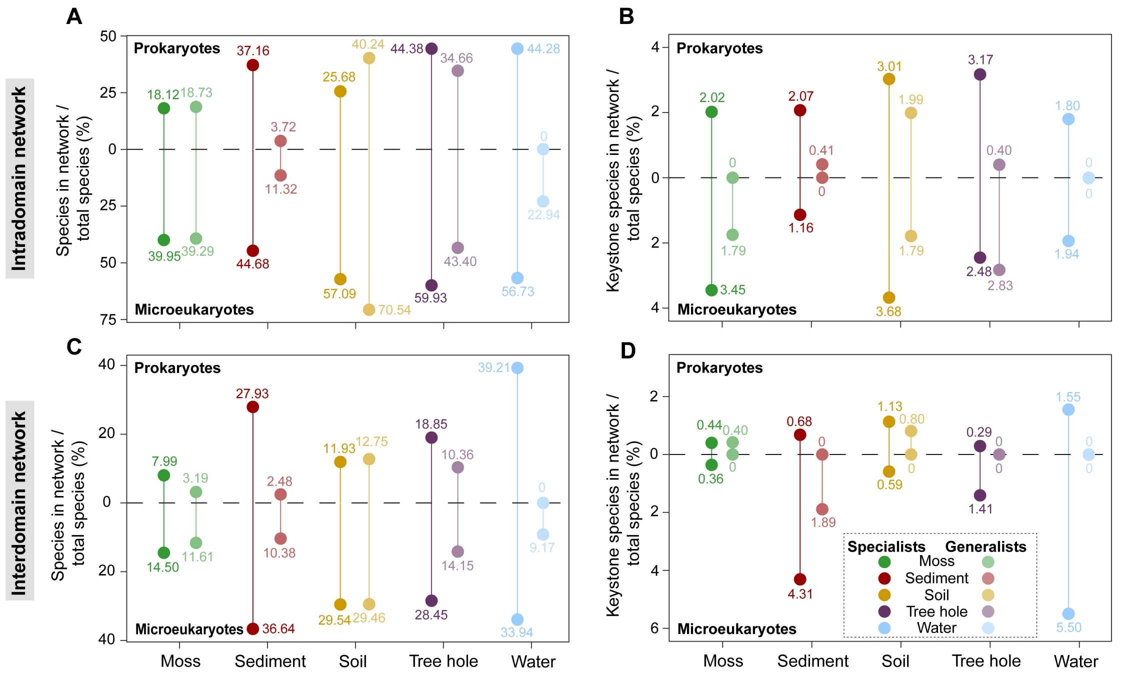

Fig S10 Percentages of habitat generalists and specialists in the microbial network. **(A)** The percentage of habitat specialists or generalists in the intradomain network from total specialists or generalists. **(B)** The percentages of identified keystone species in the intradomain network from total specialists or generalists. **(C)** The percentage of specialists or generalists in the interdomain network from total specialists or generalists. **(D)** The percentage of keystone species in the interdomain network from total specialists or generalists.

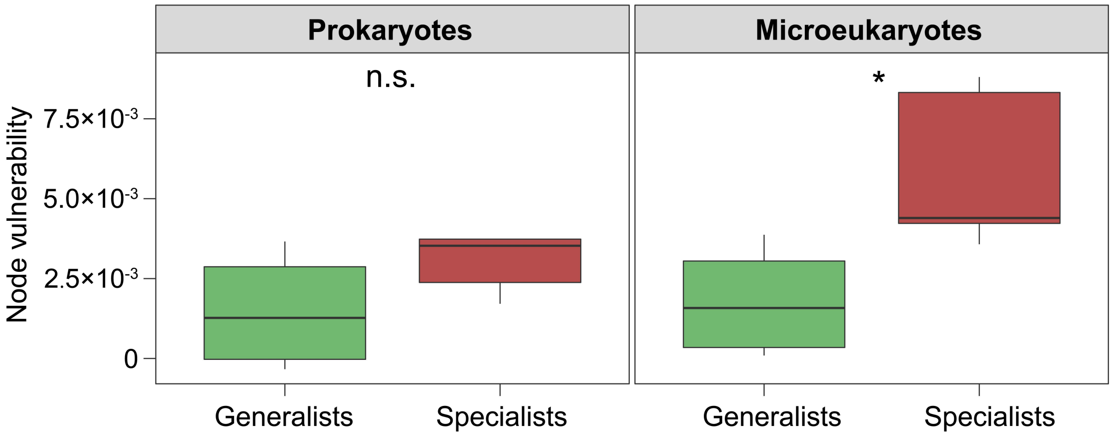

Figure S11 Vulnerability of networks measured from the contribution of the node in network to global efficiency. The significance test was performed by the Wilcoxon test. *$P < 0.05$.

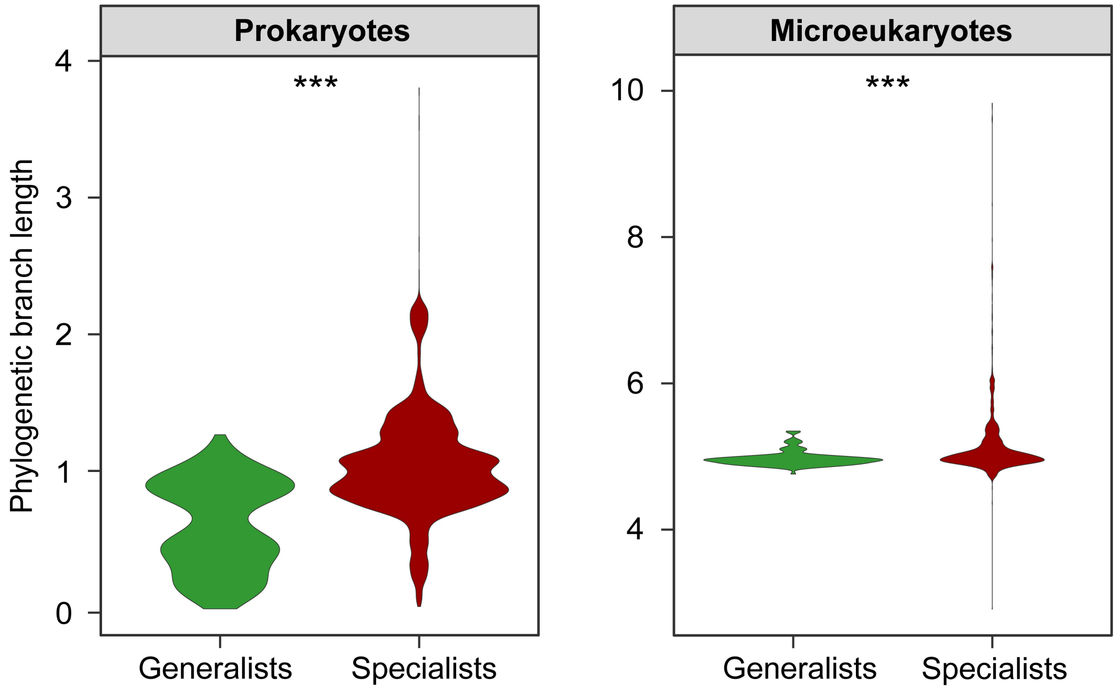

Figure S12 The phylogenetic branch length of generalists and specialists. The significance test was performed by the Wilcoxon test. ***$P < 0.001$.

Table S1 Prokaryotic zOTUs table with abundances and classifications, three niche breadth values (i.e., Levins' niche breadth, Shannon diversity, and occurrence), and assignments of generalists and specialists.
See supplementary Excel file for details.

Table S2 Microeukaryotic zOTUs table with abundances and classifications, three niche breadth values (i.e., Levins' niche breadth, Shannon diversity, and occurrence), and assignments of generalists and specialists.
See supplementary Excel file for details.

Table S3 Good's coverage of prokaryotic and microeukaryotic communities. Values are mean ± standard deviation.

Park	Habitat	Prokaryotes	Microeukaryotes
Dalunshan (DLS)	Moss	0.938 ± 0.017	0.991 ± 0.001
	Sediment	0.973 ± 0.003	0.993 ± 0.001
	Soil	0.928 ± 0.004	0.987 ± 0.003
	Tree hole	0.937 ± 0.008	0.990 ± 0.001
	Water	0.973 ± 0.004	0.989 ± 0.002
Dapingshan (DPS)	Moss	0.953 ± 0.015	0.990 ± 0.002
	Sediment	0.955 ± 0.004	0.991 ± 0.001
	Soil	0.935 ± 0.001	0.988 ± 0.002
	Tree hole	0.936 ± 0.017	0.990 ± 0.003
	Water	0.981 ± 0.002	0.991 ± 0.001
Huli (HL)	Moss	0.929 ± 0.020	0.990 ± 0.001
	Sediment	0.958 ± 0.006	0.990 ± 0.001
	Soil	0.923 ± 0.004	0.987 ± 0.001
	Tree hole	0.939 ± 0.004	0.990 ± 0.001
	Water	0.975 ± 0.004	0.989 ± 0.003
Shangli (SL)	Moss	0.926 ± 0.001	0.991 ± 0.001
	Sediment	0.939 ± 0.009	0.993 ± 0.002
	Soil	0.918 ± 0.012	0.984 ± 0.001
	Tree hole	0.959 ± 0.010	0.989 ± 0.001
	Water	0.986 ± 0.003	0.990 ± 0.001
Shuanglongtan (SLT)	Moss	0.949 ± 0.018	0.989 ± 0.001
	Sediment	0.938 ± 0.024	0.991 ± 0.001
	Soil	0.929 ± 0.005	0.986 ± 0.002
	Tree hole	0.953 ± 0.011	0.991 ± 0.001
	Water	0.979 ± 0.004	0.990 ± 0.001
Xiangshan (XS)	Moss	0.943 ± 0.008	0.990 ± 0.001
	Sediment	0.938 ± 0.029	0.990 ± 0.002
	Soil	0.943 ± 0.009	0.986 ± 0.002
	Tree hole	0.928 ± 0.018	0.990 ± 0.001
	Water	0.976 ± 0.002	0.987 ± 0.002

Table S4 Permutational multivariate analysis of variance (PERMANOVA) using Bray-Curtis dissimilarity.

Community	Group	F	R^2
Prokaryotes	Habitat (5)	12.717	**0.251*****
	Park (6)	3.742	**0.092*****
Microeukaryotes	Habitat (5)	13.674	**0.293*****
	Park (6)	3.034	**0.081*****

Boldface, statistically significant at ****P* < 0.001.

Table S5 Network properties of prokaryotic and microeukaryotic intradomain networks.

		Empirical Network									Randomized network[a]		
Community	Habitat	Total node	Generalists node (%)	Specialists node (%)	Opportunists node (%)	Total links (positive link)	R^2 of power-law	avgCC	GD	M	avgCC	GD	M
Prokaryotes	Moss	1740	2.701	25.805	71.494	10874 (96.42%)	0.982	0.417[b]	4.439[c]	0.720[d]	0.003 ± 0.001	3.216 ± 0.010	0.225 ± 0.002
	Sediment	3168	0.284	39.678	60.038	100418 (86.98%)	0.955	0.550[b]	3.159[c]	0.500[d]	0.012 ± 0.001	2.516 ± 0.002	0.094 ± 0.003
	Soil	3997	2.527	17.063	80.410	109019 (76.62%)	0.989	0.477[b]	3.286[c]	0.442[d]	0.012 ± 0.001	2.701 ± 0.003	0.098 ± 0.004
	Tree hole	3354	2.594	35.927	61.479	77279 (97.28%)	0.985	0.523[b]	3.917[c]	0.585[d]	0.011 ± 0.001	2.722 ± 0.005	0.112 ± 0.002
	Water	1346	0	63.819	36.181	62973 (94.38%)	0.235	0.716[b]	2.705[c]	0.669[d]	0.020 ± 0.001	2.088 ± 0.001	0.094 ± 0.005
Microeukaryotes	Moss	1903	2.312	35.313	62.375	28139 (80.12%)	0.967	0.492[b]	3.498[c]	0.531[d]	0.008 ± 0.001	2.747 ± 0.004	0.131 ± 0.002
	Sediment	1266	0.948	45.814	53.239	31940 (80.37%)	0.943	0.605[b]	3.076[c]	0.545[d]	0.015 ± 0.002	2.418 ± 0.004	0.106 ± 0.005
	Soil	3187	2.479	30.185	67.336	115111 (80.54%)	0.964	0.584[b]	3.079[c]	0.442[d]	0.014 ± 0.001	2.481 ± 0.002	0.097 ± 0.005
	Tree hole	1945	2.365	37.224	60.411	38607 (86.28%)	0.951	0.568[b]	3.407[c]	0.603[d]	0.009 ± 0.001	2.603 ± 0.003	0.116 ± 0.002
	Water	1838	1.360	68.390	30.250	65101 (92.79%)	0.922	0.675[b]	3.345[c]	0.622[d]	0.017 ± 0.001	2.365 ± 0.002	0.110 ± 0.004

[a] Mean ± standard deviation (SD) based on 100 randomized networks.

[b-d] Significant difference ($P < 0.05$) in average clustering coefficient, average path length, and modularity between empirical and randomized networks based on Student's t-test, respectively.

avgCC, Average clustering coefficient; GD, Average path distance; M, Modularity.

Table S6 Network properties of prokaryotic-microeukaryotic interdomain networks.

		Empirical network								Randomized network[a]			
Habitat	Community	Node	Links (positive link)	Module number	M	Cluster coefficient	Niche overlap	Robustness	FC	M	Niche overlap	Robustness	FC
Moss	Prokaryotes	206	1243 (96.54%)	35	0.680[b]	0.010	0.037[c]	0.697[d]	376.117[e]	0.380±0.004	0.033±0.001	0.695±0.002	467.254±4.601
	Microeukaryotes	257					0.044[c]	0.713	405.443[e]		0.045±0.001	0.713±0.002	500.311±5.088
Sediment	Prokaryotes	951	15869 (83.59%)	11	0.604[b]	0.023	0.048[c]	0.890[d]	2671.623[e]	0.154±0.002	0.044±0	0.889±0.001	3835.978±5.732
	Microeukaryotes	487					0.046[c]	0.852	2128.581[e]		0.044±0	0.852±0.001	2726.360±2.058
Soil	Prokaryotes	349	33123 (88.56%)	23	0.554[b]	0.011	0.036[c]	0.759[d]	967.462[e]	0.240±0.003	0.033±0	0.760±0.002	1156.964±4.794
	Microeukaryotes	531					0.038[c]	0.786[d]	1101.714[e]		0.037±0	0.784±0.001	1421.228±5.559
Tree hole	Prokaryotes	538	1916 (87.79%)	39	0.783[b]	0.004	0.044[c]	0.700[d]	669.978[e]	0.474±0.004	0.035±0.001	0.698±0.002	895.554±6.602
	Microeukaryotes	359					0.020[c]	0.675[d]	610.064[e]		0.013±0	0.676±0.003	772.148±5.120
Water	Prokaryotes	761	9761 (72.17%)	5	0.626[b]	0.024	0.072[c]	0.905[d]	1703.375[e]	0.178±0.003	0.058±0	0.903±0.001	2796.821±4.549
	Microeukaryotes	343					0.061[c]	0.859[d]	1342.619[e]		0.046±0	0.856±0.001	1853.539±1.360

[a] Mean ± standard deviation (SD) based on 100 randomized networks.

[b-e] Significant difference ($P < 0.05$) in modularity, niche overlap, robustness, and functional complementarity between empirical and randomized networks based on Student's t-test, respectively.

FC, Functional complementarity; M, Modularity.

Table S7 Identified keystone species in interdomain networks and their taxonomic annotations.

See supplementary Excel file for details.

Table S8 Prokaryotic zOTUs numbers, matched zOTUs numbers and percentages with the genome database at different levels.

Habitat	Microbial type	zOTUs number	Matched zOTUs numbers (percentages) with the genome database				
			Phylum	Class	Order	**Family**	Genus
Moss	Specialists	2479	1189 (47.96%)	1785 (72.00%)	1536 (61.96%)	**1302 (52.52%)**	706 (28.48%)
	Generalists	251	159 (63.35%)	231 (92.03%)	209 (83.27%)	**190 (75.70%)**	113 (45.02%)
Sediment	Specialists	3384	1602 (47.34%)	2331 (68.88%)	2035 (60.14%)	**1624 (47.99%)**	843 (24.91%)
	Generalists	242	154 (63.64%)	223 (92.15%)	201 (83.06%)	**184 (76.03%)**	112 (46.28%)
Soil	Specialists	2657	1264 (47.57%)	1858 (69.93%)	1655 (62.29%)	**1350 (50.81%)**	687 (25.86%)
	Generalists	251	159 (63.35%)	231 (92.03%)	209 (83.27%)	**190 (75.70%)**	113 (45.02%)
Tree hole	Specialists	2716	1269 (46.72%)	1946 (71.65%)	1638 (60.31%)	**1399 (51.51%)**	775 (28.53%)
	Generalists	251	159 (63.35%)	231 (92.03%)	209 (83.27%)	**190 (75.70%)**	113 (45.02%)
Water	Specialists	1941	963 (49.61%)	1306 (67.28%)	1170 (60.28%)	**892 (45.96%)**	423 (21.79%)
	Generalists	109	69 (63.30%)	96 (88.07%)	92 (84.40%)	**86 (78.90%)**	59 (54.13%)
Five habitats	Specialists	4210	1981 (47.05%)	2951 (70.10%)	2534 (60.19%)	**2073 (49.24%)**	1079 (25.63%)
	Generalists	251	159 (63.35%)	231 (92.03%)	209 (83.27%)	**190 (75.70%)**	113 (45.02%)

Table S9 Average genome length (Mbp) of prokaryotic specialists and generalists in each habitat.

Habitat	Indices in the genome database	Specialists (Mbp)	Generalists (Mbp)	*P* value
Moss	mean.corrected_length	4.82 ±1.62	4.97 ± 1.42	> 0.05
	mean.species.corrected_length	5.00 ±1.63	5.20 ± 1.41	> 0.05
	median.corrected_length	4.73 ± 1.59	4.92 ± 1.42	> 0.05
	median.species.corrected_length	4.93 ± 1.70	5.11 ± 1.44	> 0.05
Soil	mean.corrected_length	4.71 ± 1.56	4.97 ± 1.42	> 0.05
	mean.species.corrected_length	4.88 ± 1.60	5.20 ± 1.41	< 0.05*
	median.corrected_length	4.60 ± 1.53	4.92 ± 1.42	< 0.05*
	median.species.corrected_length	4.80 ± 1.68	5.11 ± 1.44	< 0.05*
Sediment	mean.corrected_length	4.65 ± 1.56	4.95 ± 1.40	< 0.05*
	mean.species.corrected_length	4.78 ± 1.62	5.19 ± 1.40	< 0.001***
	median.corrected_length	4.55 ± 1.55	4.91 ± 1.41	< 0.01**
	median.species.corrected_length	4.69 ± 1.67	5.11 ± 1.44	< 0.001***
Tree hole	mean.corrected_length	4.77 ± 1.62	4.97 ± 1.42	> 0.05
	mean.species.corrected_length	4.92 ± 1.64	5.20 ± 1.41	< 0.05*
	median.corrected_length	4.65 ± 1.59	4.92 ± 1.42	< 0.05*
	median.species.corrected_length	4.81 ± 1.68	5.11 ± 1.45	< 0.05*
Water	mean.corrected_length	4.45 ± 1.42	4.80 ± 1.35	< 0.05*
	mean.species.corrected_length	4.58 ± 1.53	5.01 ± 1.42	< 0.01**
	median.corrected_length	4.37 ± 1.41	4.75 ± 1.41	< 0.05*
	median.species.corrected_length	4.53 ± 1.60	4.92 ± 1.48	< 0.05*
Five habitats	mean.corrected_length	4.68 ± 1.58	4.97 ± 1.42	< 0.05*
	mean.species.corrected_length	4.82 ± 1.63	5.20 ± 1.41	< 0.01**
	median.corrected_length	4.57 ± 1.56	4.92 ± 1.42	< 0.01**
	median.species.corrected_length	4.73 ± 1.68	5.11 ± 1.45	< 0.01**

The statistic is Wilcoxon test at *$P < 0.05$, **$P < 0.01$, ***$P < 0.001$.
